# Contrast Agent Enhanced Multimodal Photoacoustic Microscopy and Optical Coherence Tomography for Imaging of Rabbit Choroidal and Retinal Vessels *in vivo*

**DOI:** 10.1038/s41598-019-42324-5

**Published:** 2019-04-11

**Authors:** Van Phuc Nguyen, Yanxiu Li, Wei Qian, Bing Liu, Chao Tian, Wei Zhang, Ziyi Huang, Arjun Ponduri, Madison Tarnowski, Xueding Wang, Yannis M. Paulus

**Affiliations:** 1 Department of Ophthalmology and Visual Sciences, University of Michigan, Ann Arbor, MI 48105 USA; 2 Department of Biomedical Engineering, University of Michigan, Ann Arbor, MI 48105 USA; 3 Department of Electrical Engineering and Computer Science, University of Michigan, Ann Arbor, MI 48105 USA; 4 IMRA America Inc, Ann Arbor, MI 48105 USA; 5 Department of Radiology, University of Michigan, Ann Arbor, MI 48105 USA; 6 Department of Ophthalmology, Xiangya Hospital, Central South University, Changsha, Hunan 410008 China; 7 NTT-Hitech Insitute, Nguyen Tat Thanh University, Ho Chi Minh, Vietnam

## Abstract

Multimodal imaging with photoacoustic microscopy (PAM) and optical coherence tomography (OCT) can be an effective method to evaluate the choroidal and retinal microvasculature. To improve the efficiency for visualizing capillaries, colloidal gold nanoparticles (AuNPs) have been applied as a multimodal contrast agent for both OCT and PAM imaging by taking advantage of the strong optical scattering and the strong optical absorption of AuNPs due to their surface plasmon resonance. Ultra-pure AuNPs were fabricated by femtosecond laser ablation, capped with polyethylene glycol (PEG), and administered to 13 New Zealand white rabbits and 3 Dutch Belted pigmented rabbits. The synthesized PEG-AuNPs (20.0 ± 1.5 nm) were demonstrated to be excellent contrast agents for PAM and OCT, and do not demonstrate cytotoxicity to bovine retinal endothelial cells in cell studies. The image signal from the retinal and choroidal vessels in living rabbits was enhanced by up to 82% for PAM and up to 45% for OCT, respectively, by the administered PEG-AuNPs, which enables detection of individual blood vessels by both imaging modalities. The biodistribution study demonstrated the AuNP accumulated primarily in the liver and spleen. Histology and TUNEL staining did not indicate cell injury or death in the lung, liver, kidney, spleen, heart, or eyes up to seven days after AuNP administration. PEG-AuNPs offer an efficient and safe contrast agent for multimodal ocular imaging to achieve improved characterization of microvasculature.

## Introduction

Pathologic microvasculature plays a crucial role in innumerable diseases causing death and major organ impairment, from stroke, cancer, and aneurysms to macular degeneration and diabetic retinopathy^1–4^. Imaging is important to diagnose and distinguish diverse pathologies to initiate early treatment. In ophthalmology, a number of imaging techniques have been explored for the evaluation of retinal and choroidal vessels of the eye such as optical coherence tomography (OCT), OCT angiography (OCTA), fluorescein angiography (FA), and indocyanine green angiography (ICGA). Each of these modalities has its own specific advantages and limitations^5,6^. For example, OCT provides structural 3D depth information of the retina with a high resolution rapidly and non-invasively. However, OCT has difficulty distinguishing choroidal neovascularization from subretinal fibrosis or hemorrhage^7^. FA is the gold standard for retinal vascular imaging. FA provides visualization of retinal circulation and leakage, but it does not indicate depth of the vasculature. ICGA can be used to image the choroid and neovascular membranes beneath the retinal pigment epithelium (RPE). OCTA allows evaluation of vasculature with depth information but does not demonstrate leakage, provides limited visualization of microaneurysms, and has a restricted field of view often with artifacts.

Photoacoustic imaging (PA) allows for high-resolution, high sensitivity, and high-contrast images of subsurface structures on targeted tissues^8^. Photoacoustic imaging is a hybrid, non-ionizing, and non-invasive imaging modality with micron-scale spatial resolution and millimeter-scale depth penetration^9,10^. It is being explored in visualizing tissue structure and function in biomedical applications, such as imaging of brain, liver, breast, joints, and eye^11–14^. In ophthalmology, several groups have investigated PA ocular imaging platforms to evaluate ocular tissues with a high depth of penetration^15–21^. Combined OCT and PA systems have been reported to be capable of imaging retinal vessels^17,22,23^, RPE cells^17,23^, and choroidal vessels^22,23^ in rodents^24–26^. Our group has successfully developed an integrated photoacoustic microscopy (PAM) and OCT system for monitoring retinal and choroidal blood vessels^27,28^ in living rabbits with high temporal and spatial resolution^27,29^. The applied light fluence for PAM was only half of the American National Standards Institute (ANSI) safety limit, holding potential for clinical translation.

Although the vasculature in the retina and choroid can be imaged with PAM without using contrast agents, the diagnostic information is limited to hemodynamic properties such as blood flow, blood volume, and oxygen saturation. The sensitivity and specificity of PAM can be improved by adding exogenous contrast agents, and the application scope of PAM can be extended from the tissue level to the molecular and cellular levels. Exogenous contrast agents for PA imaging can be classified into two groups: organic (e.g., liposomes, dyes, Indocyanine green (ICG), Prussian blue, Methylene blue, and polymeric complexes), and inorganic (e.g., gold nanoparticles, silica^30,31^, and copper sulfide nanoparticles^32,33^). Each agent has advantages and limitations. Organic agents are easily biodegradable, often rapidly cleared, and have a more established route towards clinical translation. However, the level of contrast enhancement by organic agents can be limited. Inorganic contrast agents, such as gold particles (AuNPs), have exhibited promising results in photoacoustic molecular imaging^12^. The use of AuNPs for OCT has also been studied on cells by using gold nanoshells as contrast agent to enhance OCT signal^34^. As a result of the surface plasmon resonance, AuNPs have very unique optical properties including extremely strong optical absorption and optical scattering, making them excellent candidates as multimodal contrast agents for many optical imaging modalities. AuNPs also have excellent bio-stability, photo-stability, thermal-stability, and optical tunability. By changing the size and shape, the optical absorption peak of AuNPs can be tuned throughout the visible and near infrared region^10,35–38^. Chemical synthesis of AuNP can have associated toxicities^39–42^, and thus this study uses AuNPs that were fabricated by femtosecond laser ablation on target gold in deionized water, and subsequently functionalized with polyethylene glycol (PEG)^38^. The AuNPs made by femtosecond laser ablation are ultra-pure without any chemical additives or stabilizer. PEG derivatives were used to optimize the surface properties and functionalities, enhance the solubility, stability, and biocompatibility, and prolong the systemic circulation time of AuNPs. This study evaluates the safety and utility of PEG-AuNPs in living rabbits as a multimodal OCT and PAM contrast agent for examination of retinal and choroidal microvasculature.

## Results

### Preparation and characterization of PEG-AuNPs

**Figure 1:**
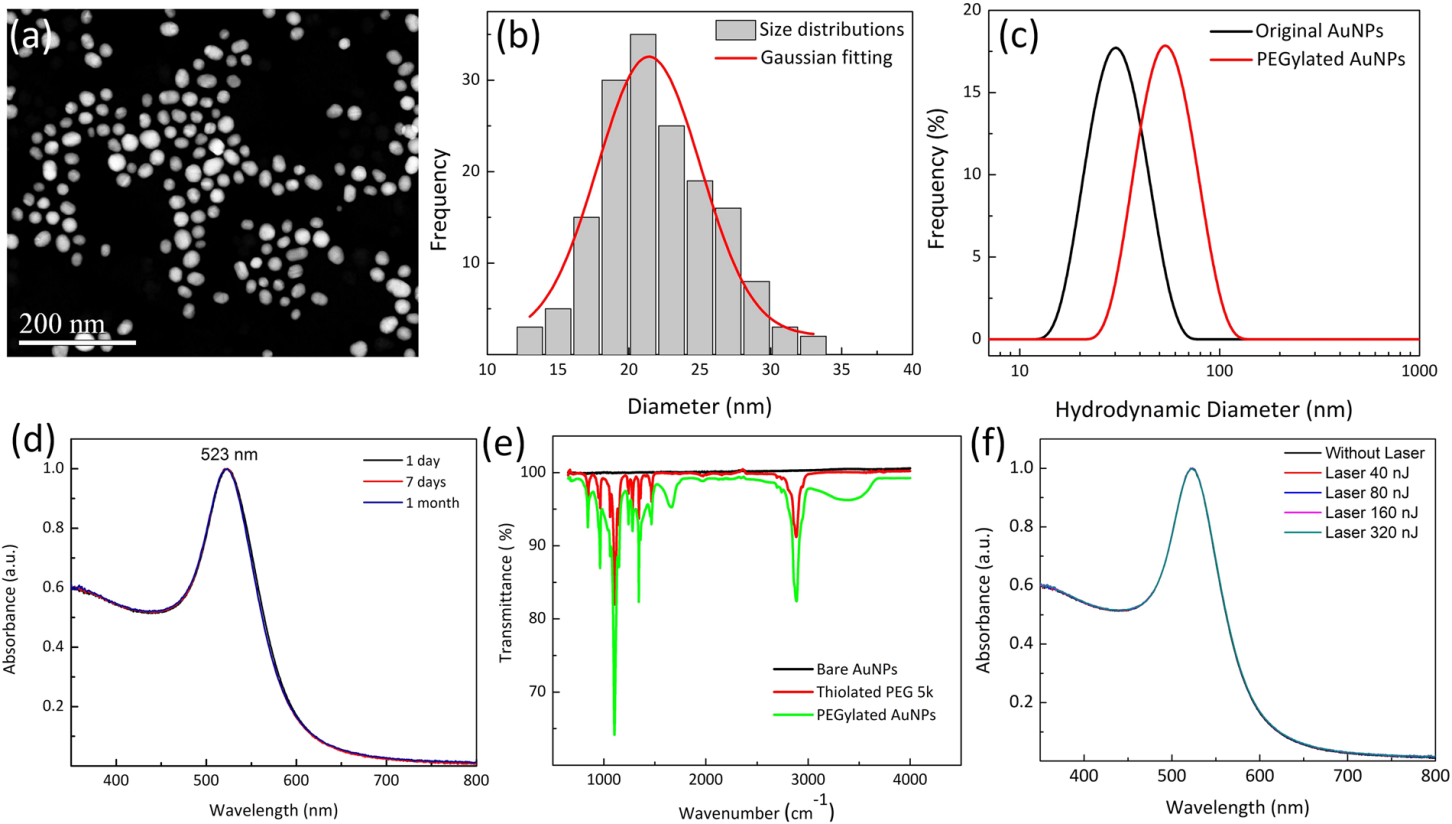
Characterization of synthesized PEGylated gold nanoparticles (PEG-AuNPs): (**a**) TEM image of 20 nm sized PEG-AuNPs (×2000, 20 kV; bar = 200 nm), (**b**) particle distribution of PEG-AuNPs estimated by TEM, (**c**) comparision of hydrodynamic sized bare AuNPs and PEG-AuNPs nanoparticles quantified by dynamic light scattering (DLS) in water, (**d**) absorption spectra of PEG-AuNPs as function of wavelength at various times (1 day, 7 day, and 1 month), and (**e**) FT-IR spectra of bare AuNPs, PEG, and PEG-AuNPs, respectively. (**f**) photostability of PEG-AuNPs after laser irradiation at different energies (40, 80, 160, and 320 nJ) for aperiod of 65 s.

Raw AuNPs were fabricated using femtosecond laser ablation of a gold target submerged in flowing deionized water^43^. This method uses tightly focused micro-joule (μJ) femtosecond laser pulses to produce nanoparticles, and the size and size distribution of generated nanoparticles can be precisely controlled by optimizing laser parameters, such as wavelength, pulse energy, pulse duration, and repetition rate^44^. During the pulsed laser ablation, AuNPs were partially oxidized by oxygen present in solution. These Au-O compounds were hydroxylated, followed by a proton loss to give a surface of Au-O^−^^45^. Therefore, the Au NPs produced using the laser ablation method are naturally negatively charged and no surfactants and stabilizing ligands are required for maintaining their colloidal stability. Colloidal AuNPs with an average diameter of 20 nm were produced and used in our experiments. Representative TEM image, hydrodynamic particle size distribution and ultraviolet–visible (UV–vis) absorption spectrum are presented in Fig. 1. The generated nanoparticles have a peak size around 20 nm and have an absorption peak at 520 nm due to localized surface plasmon resonance (LSPR). The spectral feature below 450 nm reflects gold intraband transitions since the nanoparticles were generated in deionized water without having other chemical components, such as chemical precursors, reducing agents, and stabilizing ligands, being involved in the fabrication process.

For stabilizing AuNPs under physiological conditions and preventing them from being recognized and eliminated by the human reticuloendothelial system (RES), AuNPs were surface modified with a layer of PEG molecules to improve their stability, biocompatibility, and simultaneously minimize nonspecific interactions with biological tissues by providing a hydrophilic steric barrier. The surface modification of AuNPs with a layer of PEG molecules was performed by adding into them a solution of mPEG-SH5k. The binding of PEG molecules onto the nanoparticles is possible due to strong anchoring of the thiol-gold bonds. Figure 1c compared the hydrodynamic diameters of AuNPs and PEG-AuNPs obtained by DLS measurements. The results showed that the diameter of AuNPs increased over 20 nm after the surface modification with mPEG-SH5k, confirming the result of the binding of thiolated mPEG-SH5k to the surface of AuNPs. The FTIR spectroscopy was also used to confirm the presence of mPEG-SH5k molecules on the surface of AuNPs. As demonstrated in the Fig. 1d, FTIR spectrum of PEG-AuNPs colloidal solution displayed all characteristic peaks that could be found in mPEG-SH5k but not in the AuNPs colloidal solution. In addition, the long term colloidal stability of PEG-AuNPs used in the present study up to a month was evaluated using UV-Vis absorption spectroscopy. As it were shown in the Fig. 1e, there were not any detectable decrease and red shift of LSPR around 520 nm within a month, revealing these PEG-AuNPs remained stable for at least one month. In order to evaluate the photostability of gold nanoparticles during the laser irradiation, we measured UV-vis absorption spectra of the samples after laser exposure at various laser energy (40, 80, 160, and 320 nJ) for a period of 65 s. As shown in Fig. 1(f), colloidal gold nanoparticles show high photostability against laser illumination at the wavelength of 523 nm. It was noticed that the optical spectra show almost no remarkable changes after laser irradiation even at high energy of 320 nJ.

### Biocompatibility and cytotoxicity evaluation with cells

**Figure 2:**
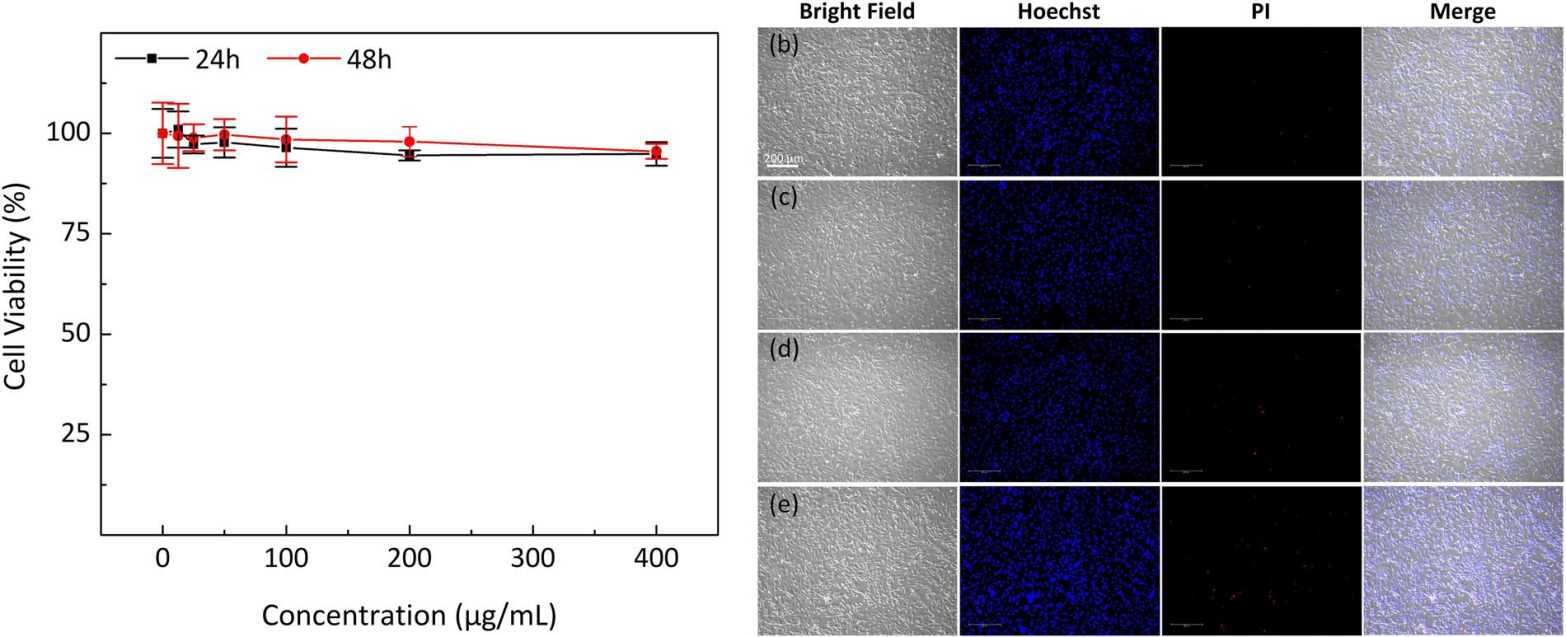
Effect of PEG-AuNPs on bovine retinal endothelial cells (BRECs) cells: (**a**) cell viability of PEG-AuNPs at different concentrations (0, 12.5, 25, 50, 100, 200, and 400 µg/mL) and incubation times (24 and 48 h). Quantified BRECs cells at various groups. Data expressed as mean ± SD (N = 3, p < 0.01). (**b–e**) fluorescence microscopy images of BRECs cells stained with Hoechst and propidium iodide (PI) after treatment with PEG-AuNPs at 0 (control), 50, 100, and 200 µg/mL, respectively. The right images are the overlaid fluorescence and bright-field images. Confocal fluorescence images of BRECs cells used to detect live/dead cell after treated with AuNPs. The fluorescence color represents the nucleic morphology of cell affected by AuNPs. Nuclei were stained with Hoechst 33342 (blue). The dead cells were observed by red staining with PI. (10; bar = 200 µm). The fluorescence images were acquired at 461 nm for Hoechst 33342, and 617 nm for PI, under excitation at 350 nm for Hoechst 33342, and 535 nm for PI, respectively.

Bovine retinal endothelial cells (BREC) were used to investigate the *in vitro* biocompatibility of the PEG-AuNPs. Figure 2(a) shows the viability of BREC cells treated with the PEG-AuNPs suspension at different concentrations and incubation times. The control group illustrated no change in cell viability for all incubation times. The survival rate of the treated BREC cells was not significantly decreased for most concentrations of PEG-AuNPs and incubation times. The cell viability was reduced slightly at the highest concentration of PEG-AuNPs during a 24-h incubation by approximately 5.1% compared to control cells (untreated) (survival rate = 100% for control vs. 94.9% for 400 µg/mL of PEG-AuNPs). This is consistent with the biocompatibility of polymer coated gold nanoparticles studies^46^, which demonstrated that PEG-AuNP did not show significant toxicity, even at the concentration of 1 mg/mL. Similarly, the cell viability slightly decreased with increasing PEG-AuNPs concentrations after a 48-h incubation. The cells treated with PEG-AuNPs suspension at the highest concentration of 400 µg/mL still exhibited 94.9% and 95.5% cell viability after 24 h and 48 h of incubation. These results indicate that PEG-AuNPs demonstrate excellent biocompatibility and are suitable for *in vivo* studies, which agrees with previous studies^47^.

To observe the apoptotic activity of PEG-AuNPs, BREC cells were treated with PEG-AuNPs at different concentrations (i.e. 50, 100, and 200 µg/mL) for 4 h. After treatment, the treated cells were washed with PBS and stained with Hoechst 33342 and propidium iodide (PI) to detect cell apoptosis and necrosis. Typically, the live and dead cells present variations in nuclear morphology as shown in Fig. 2(b–e). The blue emission is from the Hoechst dye, which was used to stain the nucleus of viable BREC cells, and the red color from PI staining indicates dead cells. The control group displayed normal nuclear contour with normal cellular morphology (PI staining), indicating that cells were viable and that cytotoxicity was insignificant (Fig. 2(b)). Similarly, the treated cells with various concentrations of nanoparticles also exhibited few marked changes in the cellular morphology based on PI staining (Fig. 2(c–e)). Importantly, the death of most cells as shown in Fig. 2(e) demonstrated the potential enhancement of toxic effects resulting from elevated PEG-AuNPs concentration (i.e., 200 µg/ml).

### *In vitro* photoacoustic microscopy

**Figure 3:**
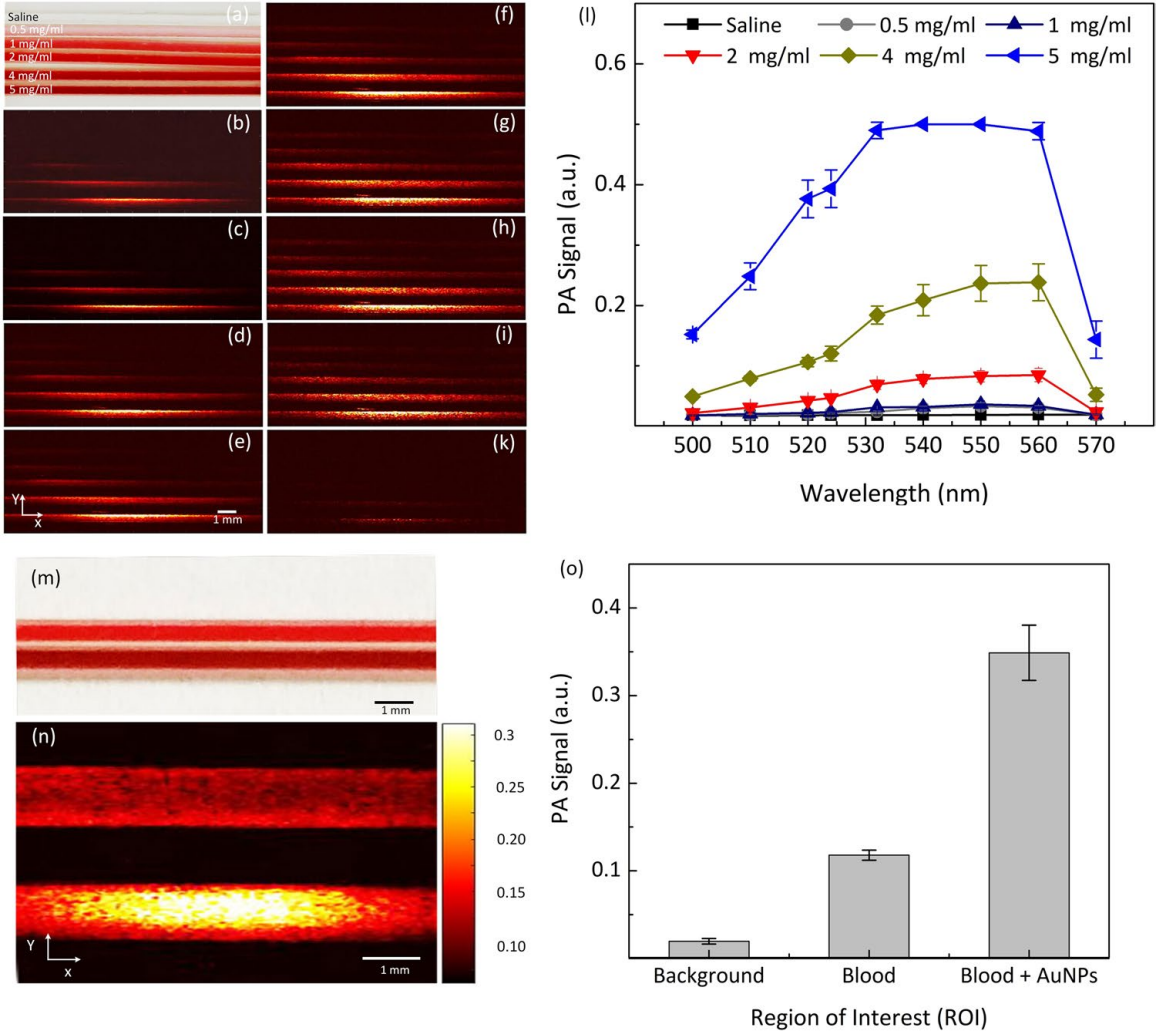
Quantitative analysis of photoacoustic (PA) responses of PEG-AuNPs: (**a**) photographs of phantom filled with AuNPs at various concentrations (0 (saline), 0.5, 1, 2, 4, 5 mg/mL). (**b–k**) corresponding PA images acquired at different wavelengths, ranging from 500 to 570 nm, under laser irradiation at an energy of 28 nJ. (l) estimated PA signals as function of PEG-AuNPs wavelength and concentration. The PA signal was gradually increased with increasing of wavelength and concentration of AuNPs. (**m**) photographs of phantoms containing blood and mixed blood and AuNPs (5 mg/ml) at ratio of 1:1, and (**n**) corresponding PA images. The sample filled with mixed blood and AuNPs showed higher contrast in comparison with the sample filled with blood only. (**o**) comparison PA signals measured at various region of interests (ROI) on the acquired PA image (i.e., background, blood, and mixed blood and AuNPs). The PA signal of the mixed blood and AuNPs sample was significantly increased and exhibited 3-fold, and 17.5-fold higher than the blood sample and background, respectively (i.e., PA signal = 0.35 ± 0.03, 0.12 ± 0.01, and 0.02 ± 0.003 for blood + AuNPs, blood, and background, respectively).

Figure 3(a) presents a photograph of phantoms poured with different concentrations of PEG-AuNPs (0.5, 1, 2, 4, and 5 mg/mL). Figure 3(b–k) exhibits the corresponding PAM images acquired at various laser wavelengths ranging from 500 to 570 nm with a 10 nm increment from the samples in Fig. 3(a). All samples were clearly visible with high signal at a concentration of 5 mg/mL. In contrast, the samples with concentrations of 0.5 and 1 mg/mL demonstrated minimal signal. In addition, due to the transducer’s directivity pattern, the PA signal was slightly different at the center of FOV when compared with the other regions within the selected scanning areas. Figure 3(l) shows the spectroscopic PA signal measurements to determine the optimal PAM wavelength for identifying the margin of blood vessels after administration. Overall, the spectroscopic measurements qualitatively presented that the wavelengths from 532 to 560 nm achieved the maximum PA signals from the samples in comparison with other wavelengths (Fig. 3(b–k)). At the highest concentration of nanoparticles, quantitative measurements in Fig. 3(l) also illustrates that the PA signals rapidly increased with the wavelength and then reached the peak values at 532–560 nm (i.e., 0.49 ± 0.01, 0.50 ± 0.01, 0.50 ± 0.01, and 0.49 ± 0.01 for 532, 540, 550, and 560 nm, respectively). Then, the PAM signal decreased to 0.14 ± 0.03 at 570 nm. It was noted that the PAM signal at 532 nm was 3.3 and 3.5 times strong as those at shorter or longer wavelengths (i.e., 0.49 ± 0.01 at 532 nm vs. 0.15 ± 0.01 at 500 nm and 0.14 ± 0.03 at 570 nm).

Two samples poured with blood and mixed blood and aqueous suspensions of PEG-AuNPs (concentration: 5 mg/mL) were quantitatively compared in terms of image contrast acquired at 532 nm (Fig. 3(m)). The sample filled with PEG-AuNPs was clearly visible with high image contrast, whereas the blood sample has less image contrast (Fig. 3(n)). Compared with the background, the PA image contrast of PEG-AuNPs was estimated to be 16.5 ± 2.9, i.e., mean acoustic amplitude ± standard deviation (i.e., PA signal = 0.35 ± 0.03 and 0.02 ± 0.003 for blood + AuNPs and background, respectively), which was approximately twice as high as that of blood alone (i.e., 0.12 ± 0.01), as shown in Fig. 3(o)).

### ***In vivo*** PAM/OCT for choroidal and retinal blood vessels detection

**Figure 4:**
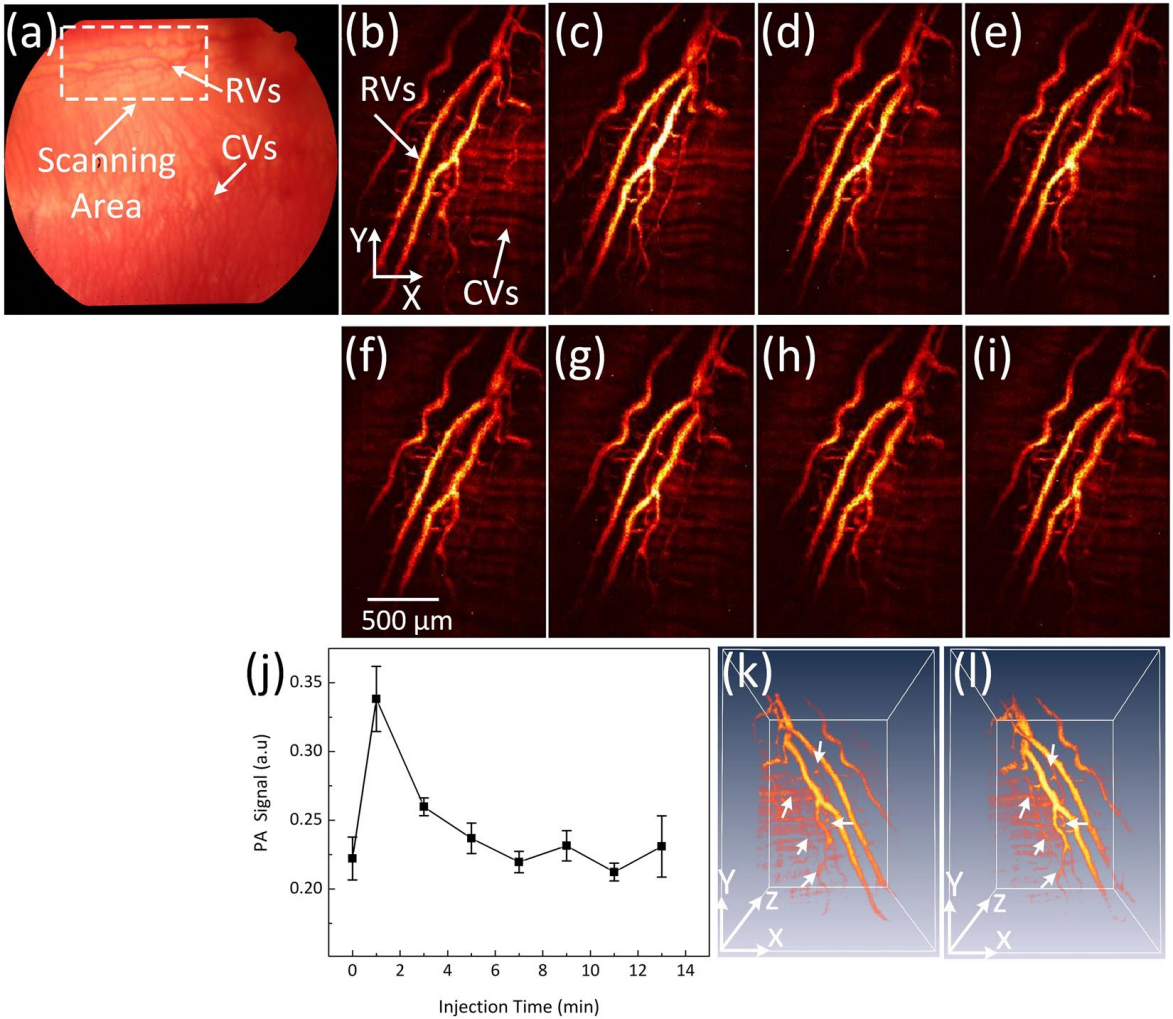
PA imaging of retinal vessels injected with PEG-AuNPs at concentration of 2 mg/ml: (**a**) color fundus image of retina. White arrow indicates the position of retinal and choroidal blood vessels. The white dotted rectangle shows the selected scanning region. (**b–i**) corresponding MIP PAM images before and after injection of PEG-AuNPs. The MIP PAM images show clearly structure of individual retinal blood vessels. Particularly, the PAM image after I.V injection shows higher contrast in comparison to the PAM image before injection. (**j**) comparison of maximum PA signals at different times on rabbit retinal vessel before and after injection of PEG-AuNPs (*p < 0.001 and N = 4). The PA signal rapidly increased after injection from 0.22 ± 0.02 (a.u.) to 0.34 ± 0.02 (a.u.). Then, the PA signal gradually decreased as time passed. (k) and (l) 3D rendering PAM image pre- and post-injection (see Visualization 1).

The capability of PEG-AuNPs as a PAM contrast agent was investigated in living rabbits (N = 16) using a multimodal PAM and OCT imaging system. The PAM imaging system operated at a wavelength of 532 nm, which is close to the optical absorption peak of PEG-AuNPs and was based on the phantom results from above. PEG-AuNPs created a strong photoacoustic signal upon exposure to laser light. Retinal and choroidal vessel imaging was acquired on rabbits before and after a single dose injection of 0.8 mL PEG-AuNPs at 2 and 5 mg/mL. Figure 4(a) presents a color fundus of the rabbit eye before *in vivo* PAM imaging. The white dotted rectangle represents the scanned areas, whereas the white dotted arrows indicate the position of the retinal vessels. The retinal vessels without injection were used as a control. The retinal vessels before and after the injection were acquired and reconstructed in both 2D and 3D. Figure 4(b) show the corresponding maximum intensity projection (MIP) of PAM images of the retinal vessels displayed in Fig. 4(a) before the administration of PEG-AuNPs; the image presents the vascular structures. Figure 4(c–i) depict MIP rendering of the data at 1, 3, 5, 7, 9, 11, and 13 minutes after PEG-AuNP injection. Figure 4(c–i) demonstrate that individual retinal microvasculature was visualized. The diameter of the major retinal vessels of the medullary rays was estimated to be 70 to 200 µm, which is consistent with previous anatomic data^48–50^. In addition, the rabbits injected with PEG-AuNPs achieved a higher photoacoustic signal compared with the control animals not injected with PEG-AuNPs (Fig. 4(b–i)).

Figure 4(j) illustrates quantitative measurement of the photoacoustic signal from Fig. 4(b–i) as a function of injection time (up to 13 min) to evaluate the dynamic contrast behavior of PEG-AuNPs. The quantified photoacoustic signal within retinal blood vessels increased immediately after PEG-AuNP injection from 0.22 ± 0.02 a.u. to 0.34 ± 0.02 a.u., p < 0.001. The PAM signal decreased over time. The PA signal enhancement within the retinal vessels injected with PEG-AuNPs (i.e., (PA_Postinjection_/PA_Preinjection_ −1) × 100) exhibited a 52% increase over that of the retinal vessels without PEG-AuNPs injection. To compare PAM signals within blood vessels to adjacent tissues, PAM signals were measured at various regions in the acquired PAM image (i.e., retinal vessels, myelinated nerve fibers, and choroidal vessels). The PAM image contrast of the post-injected retinal vessel was approximately 16 times higher than signal from the myelinated nerve fibers (i.e., photoacoustic signals = 0.34 ± 0.02 (a.u.) for injected retinal vessel vs. 0.02 ± 0.01(a.u.) for myelinated nerve fibers, p < 0.001). In contrast, the PAM contrast of retinal vessel before injection was estimated to be 10.00 ± 1.25 (i.e., photoacoustic signals = 0.22 ± 0.02 (a.u.) for pre-injected retinal vessel vs. 0.02 ± 0.01(a.u.) for myelinated nerve fibers, p < 0.001). Similarly, the PAM contrast of retinal vessels before and after injection was 3.4 and 7.5-fold higher than that of choroidal vessels. However, the myelinated nerve fiber area shows no change in the photoacoustic signal. In addition, to demonstrate the ability to visualize the enhanced contrast, the photoacoustic images pre-and post-injection of nanoparticles was subtracted. As shown in Figure S2(A), the PAM signal from the hemoglobin and hemoglobin with pure PEG-AuNPs are suppressed, revealing only the areas with the PEG-AuNPs. Figure 4(k,l) present the pre-injection and the post-injection (one minute) three-dimensional (3D) images of the vessels reconstructed from sequences of B-scan cross-sectional images. The branching network of retinal microvessels was better visualized after injection of the nanoparticles (white arrows), indicating the capacity of PEG-AuNPs to enhance image contrast in tissue.

**Figure 5:**
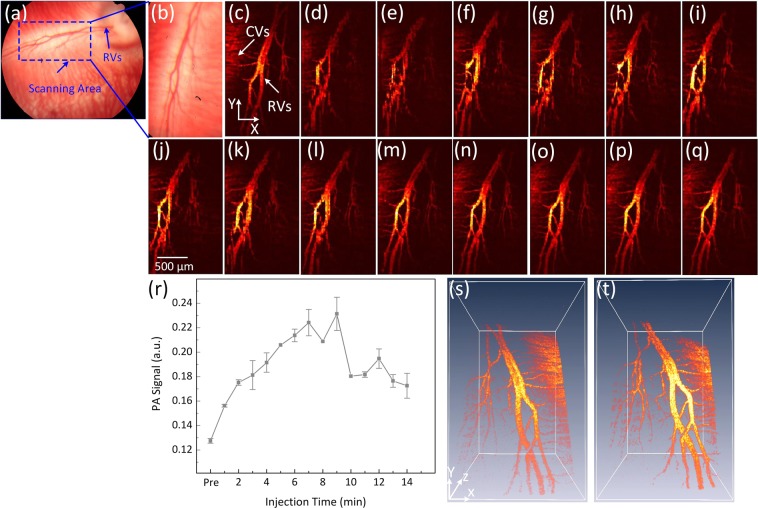
PA imaging of retinal vessels injected with PEG-AuNPs at concentration of 5 mg/mL: (**a**) color fundus image of retina, showing retinal vessels that originate from the optic nerve. The blue dotted rectangle shows the scanning area, (**b**) close-up of the retinal vessels in the dotted rectangle box in (**a**). (**c–q**) corresponding MIP PA images of retinal vessels before and after injection of PEG-AuNPs, (r) PA signal as a function of injection time on rabbit retinal vessel before and after injection of PEG-AuNPs (*p < 0.05 and N = 3), (**s**) and (**t**) 3D volumetric rendering of the PAM image pre- and post-injections, respectively (see Visualization 2). The 3D rendering image after injection showing retina with high contrast. In addition, retinal and choroidal vessels are located at different depths.

To examine the sensitivity as well as the dynamic contrast performance of PEG-AuNPs *in vivo*, the concentration of PEG-AuNPs was increased to 5 mg/mL. Figure 5(a) shows the fundus image of retinal vessels (RVs) before injection of PEG-AuNPs. Figure 5(b) exhibits the close-up of the retinal vessels in the dotted rectangle box in Fig. 5(a). Figure 5(c) shows the corresponding MIP of PAM images of the control displayed in Fig. 5(a) before the injection of PEG-AuNPs. Figure 5(d–q) show the PAM image acquired every minute over the period of 14 minutes after the injection. PEG-AuNPs caused an increase of the PAM image contrast. When the concentration of PEG-AuNPs was changed to 5 mg/mL, the photoacoustic signal increased up to 82% compared to the signal before injection as shown in Fig. 5(r). Then, the PAM signal decreased over time. Nevertheless, the PAM signal within RVs after a 14 min injection still exhibited a 35% higher PA signal than RVs without the injection of PEG-AuNPs (i.e., PAM signals = 0.13 ± 0.01(a.u.) for pre-injection RVs vs. 0.17 ± 0.01 (a.u.) for post-injection RVs, p < 0.05). The distribution of PEG-AuNPs after injection is visualized further using a subtraction algorithm (Figure S2(B)). It was also noted that the images after injection (d-l) were a little blurred in comparison with the ones in (c) and (m-q) due to the effect of median filter applied to remove someartifact occurring during the data acquisition. Figure 5(s,t) presents a 3D structure of the retinal vessels before and after injection (10 minutes), and demonstrate improved visualization of the RVs and microvasculature after injecting PEG-AuNPs.

**Figure 6:**
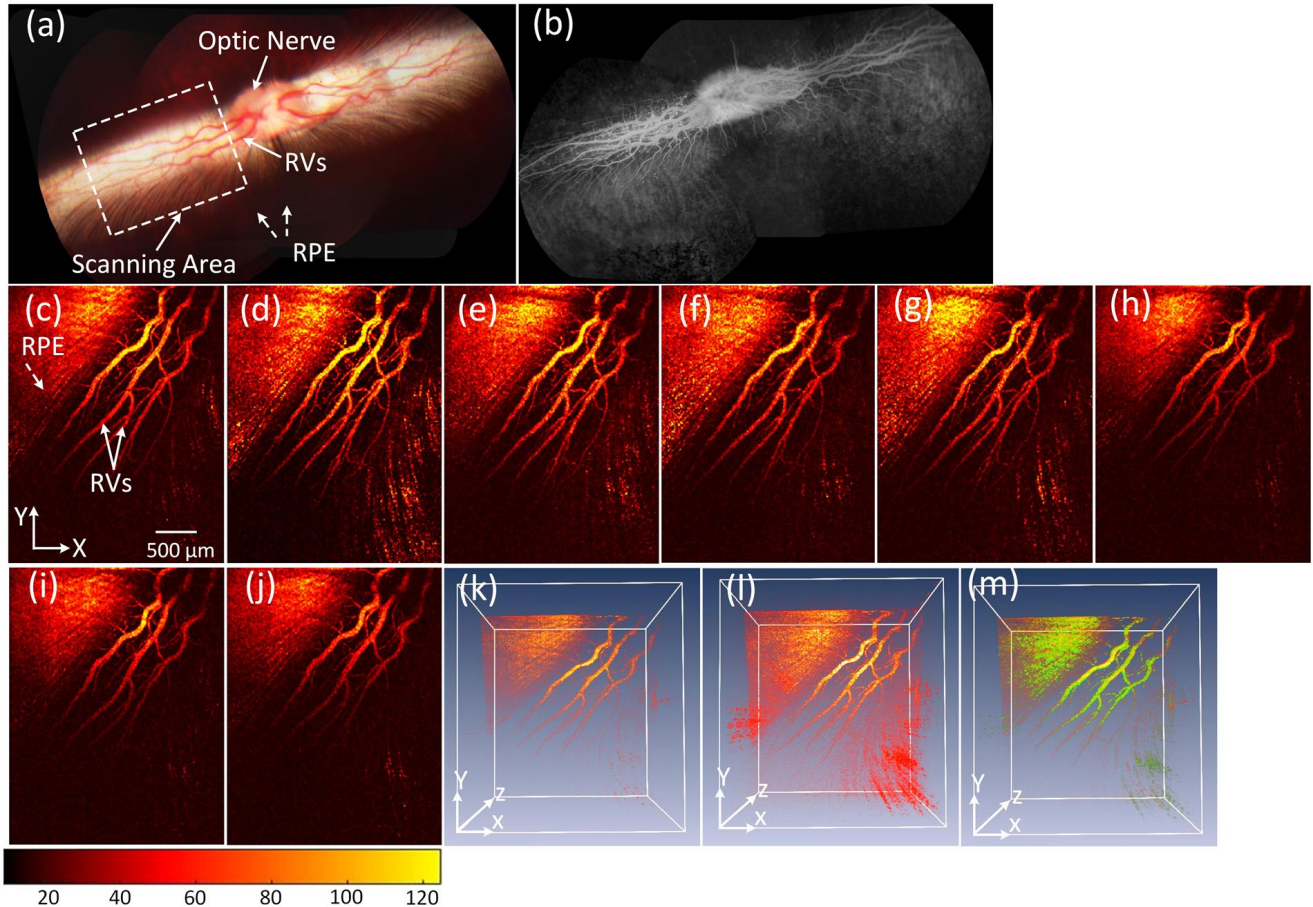
PA imaging of retinal vessels injected with PEG-AuNPs at a concentration of 5 mg/mL in pigmented rabbits: (**a**) color fundus image of the retina, showing retinal vessels (RVs), optic nerve, and retinal pigment epithelium (RPE). The white dotted rectangle shows the scanning area, (**b**) fluorescein angiography (FA) image. (**c-j**) corresponding MIP PA images of retinal vessels before and after injection of PEG-AuNPs, (**k**) and (**l**) 3D visualization of the PAM data pre- and post-injections, respectively. The 3D rendering image after injection showing retina with high contrast. In addition, retinal and choroidal vessels are located at different depths. (**m**) substraction and overlay 3D image. Pseudo green color reveals the signal enhancement after PEG-AuNPs administration.

The potential of PEG-AuNPs for enhancement of PA signal was also performed on Dutch Belted pigmented rabbits *in vivo* in order to evaluate whether melanin would affect the PA signal amplitude and quality of the acquired images. Figure 6(a) shows a color fundus photography of a pigmented rabbit. Pigmented rabbits demonstrate a melanin-containing retinal pigment epithelium with a white medullary ray. The white dotted rectangle represents the scanned areas. The white arrows illustrate the location of the retinal vessels (RVs) and optic nerve whereas the white dotted arrows represent the position of the retinal pigment epithelium (RPE). Figure 6(b) shows the fluorescein angiography (FA) image. The FA image demonstrates improved visualization of retinal vasculature. Figure 6(c) shows the corresponding MIP PAM image acquired along the dotted rectangle shown in Fig. 6(a) before the injection of AuNPs. Figure 6(d–j) exhibit PAM images obtained at 1, 3, 5, 7, 9, 11, and 13 minutes after the injection of PEG-AuNPs (0.5 ml, 5 mg/ml). Quantitative measurement of the photoacoustic signal (Figure S3) demonstrated that the photoacoustic signal increased up to 62% at 1 min post-injection compared to the signal before injection (i.e., PAM signal = **0.13** **±** **0.002**(a.u.) for pre-injection RVs vs. 0.22 ± 0.022(a.u.) for post-injection RVs, p < 0.05). Then, the PAM signal gradually decreased over time. Figure 6(k,l) exhibit the pre-injection and the post-injection (one minute) three-dimensional (3D) visualization of the PAM data. The retinal vasculature shows higher contrast and retinal capillaries were better visualized after injection of the nanoparticles (white arrows), indicating the capacity of PEG-AuNPs to enhance image contrast in tissue. This demonstrates not only that PEG-AuNPs enhanced the PA contrast of the retinal vessels in albino rabbits but also that the PEG-AuNPs possibly elevates the PA amplitudes in pigmented rabbits. In addition, image subtraction and overlay were performed to visualize the enhanced contrast as shown in Fig. 6(m). Pseudo green color was used to indicate the PA signal enhancement after injection of PEG-AuNPs.

**Figure 7:**
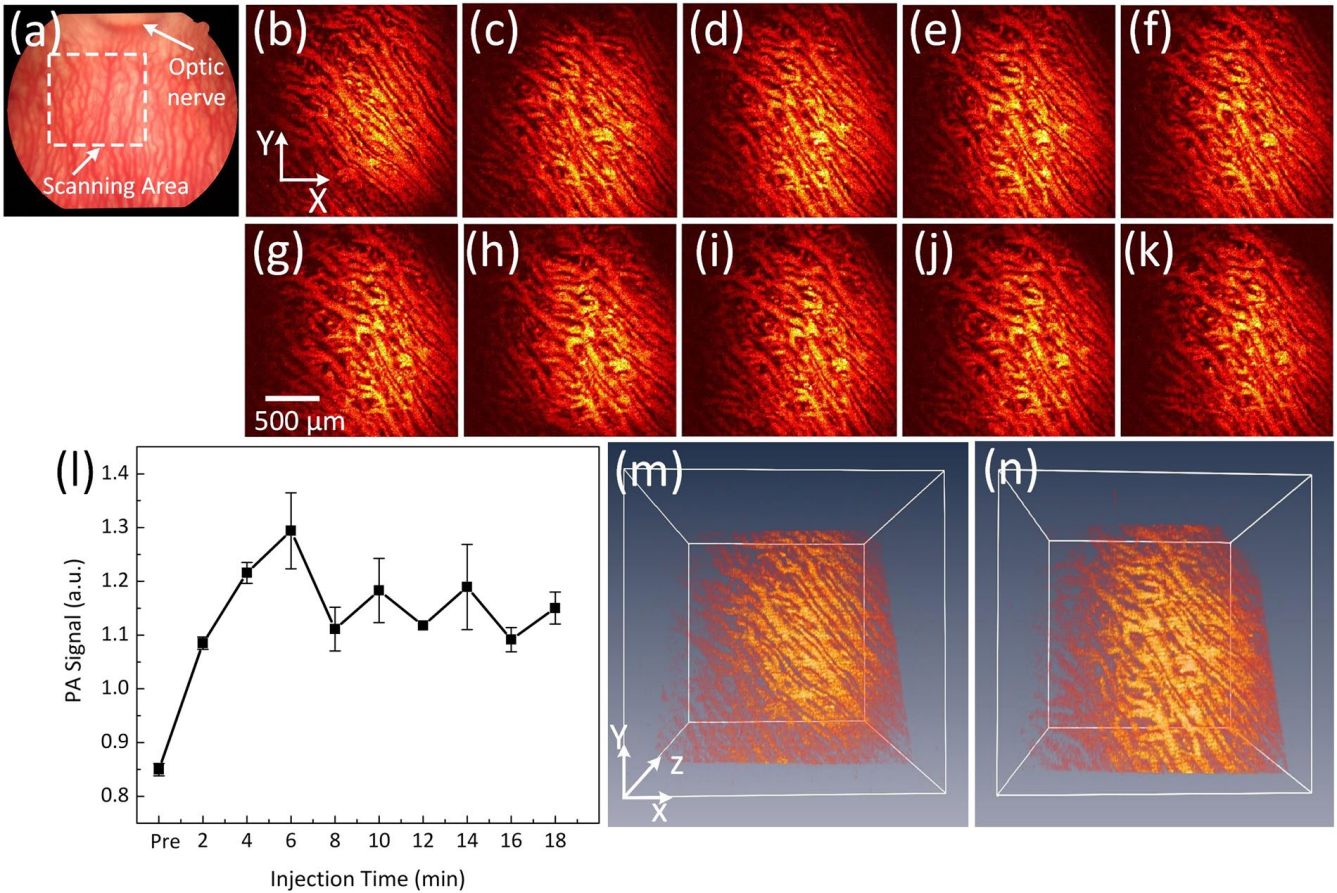
PAM of choroidal blood vessels in rabbits: (**a**) fundus photograph showing rabbit choroidal vessels. The white dotted square box represents the scanning area, (**b–k**) corresponding MIP of PAM signals of choroidal vessels (CVs) before and after injection of PEG-AuNPs (400 µl, 2 mg/ml), (**l**) comparison of PA signals at various injection times. The PA signal was gradually increased after injection of nanoparticles and reached peak value at a time of 6 min. Afterwards, the PA signal slightly decreased as time passed. (**m**) and (**n**) 3D image reconstruction of CVs pre- and post-injections, respectively (see Visualization 3).

The multimodal imaging system was also employed to monitor choroidal blood vessels (CVs) in rabbits. The choroidal vessels are clearly visible in the rabbit fundus inferior to the optic nerve as shown in Fig. 7(a). Figure 7(b) displays the acquired PAM image of CVs along the dotted square box in Fig. 7(a) before injection. PAM visualizes individual CVs with high resolution. In contrast, Fig. 7(c–k) illustrate the PAM image of CVs after injection of PEG-AuNPs with high contrast. As expected, the CVs containing PEG-AuNPs produces a much stronger photoacoustic signal. This effect is quantified and plotted in Fig. 7(l) to confirm the enhancement of photoacoustic signals from the administration of PEG-AuNPs. The photoacoustic signal within choroidal blood vessels rapidly increased after the injection and reached the strongest signal after 6 min in comparison with the PA signal before injection (i.e., PAM signals = 1.29 ± 0.07 (a.u.) for post-injection vs. 0.85 ± 0.01 (a.u.) for control, p < 0.001). Then, the PAM signal declined and reached a 35% higher PA signal than CVs without injected PEG-AuNPs at 18 minutes (i.e., PAM signals = 0.85 ± 0.01 (a.u.) for pre-injection CVs vs. 1.15 ± 0.03 (a.u.) for 14 min post-injection CVs, p < 0.05). Choroidal vessels injected with PEG-AuNPs revealed a 52% higher photoacoustic signal than choroidal vessels without injected PEG-AuNPs. Figure 7(m–n) show a 3D rendering of the choroidal vessels before and after injection (6 minutes), demonstrating improved visualization of the choroidal microvasculature.

**Figure 8:**
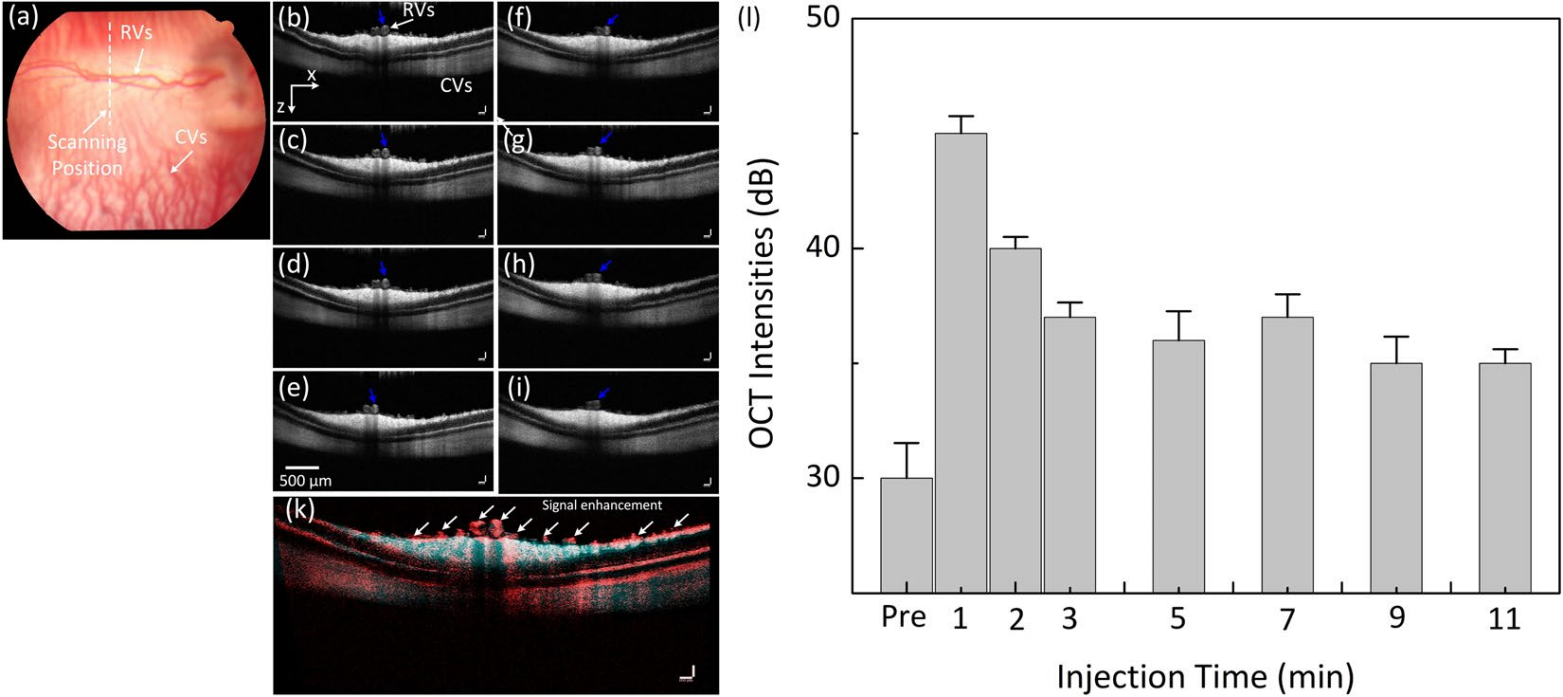
OCT image of retinal and choroidal vessels on rabbits: (**a**) Fundus photograph of retinal vessels, (**b–i**) corresponding OCT image acquired along the dotted line in (**a**) before injection and at various time after I.V. injection of AuNPs at concentration of 5 mg/mL. The OCT images show not only the structure of retinal and choroidal vessels before and after IV injection of PEG-AuNPs, but also the position of retinal and choroidal vessels at different depths. The retinal vessels and capillaries were clearly observed after injection due to high contrast. (**k**) post-OCT image subtraction and overlay of retinal vessels before and after intravenous administration of 0.8 ml AuNPs at the concentration of 5 mg/mL. The subtracted image showed the enhanced OCT intensity signal after injection. White arrows indicate the signal enhancement. (l) comparison of OCT intensity signal at various treatment times (*p < 0.05 and N = 3). The OCT intensity signals were rapidly increased from 31.67 ± 1.53 dB to 45.77 ± 0.75 dB after injection, and gradually reduce later on.

To demonstrate the capability of PEG-AuNPs for contrast-enhanced OCT, the OCT signals of PEG-AuNPs were immediately examined on the rabbit following the injection of the PEG-AuNPs and compared with the OCT signals from the rabbit without injection. Figure 8(b–i) show OCT images acquired along the dotted lined depicted on the color fundus image in Fig. 8(a) before and after injection of PEG-AuNPs at various times. The white arrows indicate the location of retinal blood vessels and capillaries adjacent to the optic nerve head. The blue arrows show the region of interest (ROI) for OCT intensity quantification. These OCT images show that choroidal vessels, retinal vessels and optic never fiber are located in different layers. The thickness between retinal and choroidal blood vessels was estimated to be 200 ± 1.8 µm. This results well agrees with the previous reports by Tian *et al*.^27^ and Kashani *et al*.^51^. Image subtraction analysis enabled detection of strong signal following the injection as shown in Fig. 8(k). The image subtraction was produced by subtracting the pre-contrast OCT image acquired prior to injection of the nanoparticles from the post-contrast OCT image acquired 1 minute after injection of the nanoparticles (Figure S4). The retinal vessels and the capillaries were clearly observed (pseudo-red color) with high contrast. Figure 8(l) shows the spectral OCT intensities as a function of injection time. The OCT intensities were measured at a region of interested This profile exhibited that the rabbit injected with AuNPs rapidly increased and exhibited ~45% greater OCT intensity than control (i.e., OCT intensity signal = 31.67 ± 1.53 dB for control vs. 45.77 ± 0.75 dB post-injection, p < 0.01).

### ***In vivo*** toxicity analysis and biodistribution of AuNPs

**Figure 9:**
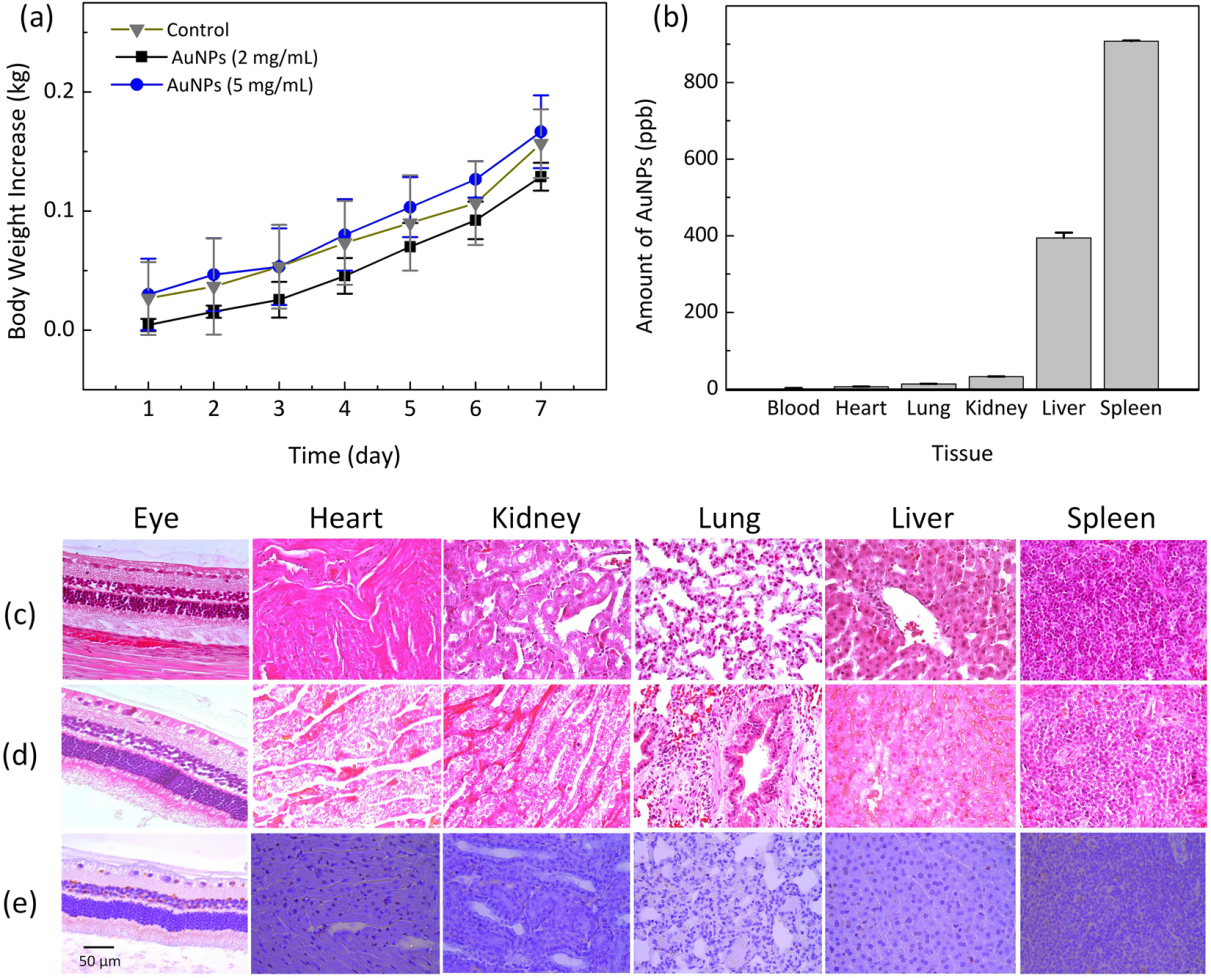
Histological and toxic analysis of PEG-AuNPs after intravenous administration: (**a**) growth of body weight as function of time after various treatment conditions: rabbits untreated with PEG-AuNPs (control), rabbit groups treated with PEG-AuNPs at 2 mg/mL, and at 5 mg/mL. The body weight was measured every day over a period of 7 days after each treatment. The body weight was gradually increased for all treatment groups, and no difference was recognized between the treated and untreated groups, indicating that the PEG-AuNPs at the applied doses are not toxic. Data expressed as mean ± SD (N = 3, p < 0.01). (**b**) quantitative analysis of amount of AuNPs accumulated in tissues by using ICP-MS method. Most AuNPs were accumulated in the liver and spleen. In contrast, very few of AuNPs were detected in blood stream. Data expressed as mean ± SEM (N = 3). (c-d) H&E image of various tissues obtained from two different groups: control (**c**), and treated with 5 mg/mL of PEG-AuNPs (**d**), the histological change and apoptosis cell death in the treated tissue were assessed through TUNEL staining assay (**e**). Note that brown color indicates the position of apoptotic cells. No significant pathological changes can be observed in the spleen or liver treated with PEG-AuNPs at a dose of 5 mg/mL. (40; bar = 50 µm).

To evaluate possible toxicity of AuNPs application, the body weights of all treated animals were monitored every day to reflect the condition of treated animals^10,52^. Generally, treatment-induced toxicity is associated with a decline in weight. Figure 9(a) shows the quantitative evaluations of animal body weight for the 7 days following treatment. Overall, the body weight of the rabbits gradually increased during the 7 days after different treatment conditions, implying that systemic toxicity was minimal in the groups. Accordingly, *in vivo* AuNPs-assisted PAM/OCT was associated with minimal adverse effects and complications.

Figure 9(b) shows the biodistribution of AuNPs in various organs. Minimal AuNPs were detected in the blood circulation after 7 days. AuNPs accumulated primarily in the liver and spleen, which is consistent with previous studies^53,54^. The spleen showed a 2.3-fold higher accumulation of AuNP than the liver. Less pronounced AuNP accumulation was noted in the lung and kidney.

To further investigate the biosafety of PEG-AuNPs, a standard histological staining with hematoxylin and eosin on lung, liver, kidney, spleen, heart, and eyes was performed at 7 days post-injection (Fig. 9(c,d)). As shown in Fig. 9, histological images showed normal cellular and tissue morphology, indicating that cells were not affected by PEG-AuNPs. In addition, TUNEL assay was also employed to further evaluate the toxicity to organs, especially the liver and spleen. As shown in Fig. 9(e), no significant TUNEL staining can be observed in any of the tissues treated with PEG-AuNPs, including the spleen and liver, demonstrating the safety and lack of cellular toxicity with the presence of AuNPs.

## Discussion

Gold nanoparticles (AuNPs) have been explored as a multimodal contrast agent for a wide variety of biomedical applications including imaging^55–58^. AuNPs have both strong optical absorption and strong optical scattering in the visible to near-infrared (NIR) spectral region^12,58,59^. Hence, AuNPs can be applied as the contrast agent for both OCT and PAM imaging of the eye. The current study is the first to investigate systemically administrated PEG-AuNPs to enhance OCT and PAM multimodality imaging of individual retinal and choroidal blood vessels in rabbit eyes *in vivo*.

Several studies have demonstrated that contrast agents can improve the effectiveness of ophthalmological disease diagnosis^60–62^. Lapierre Landry *et al*. reported that the use of the targeted AuNPs contrast agents could increase the contrast of certain layers of the retina on OCT images^62^. The current study demonstrates that AuNPs can increase PAM signals of the blood vessels by 52% (2 mg/mL) and 82% (5 mg/mL) in comparison with the control. This result suggests that PEG-AuNPs could perform as an efficient contrast agent to improve PAM imaging for the evaluation of individual retinal and choroidal microvasculature, which can be important in diseases such as macular degeneration and diabetic retinopathy. Interestingly, the AuNP signal enhancement peaks at different times between retinal and choroidal blood vessels, with retinal vessels peaking at 1 minute and choroidal vessels peaking at 6 minutes post-injection. We hypothesize that this is due to the significant difference between the vessels, namely that retinal vasculature has a blood-retinal barrier akin to the blood-brain barrier with tight junctions preventing the egress of AuNP, which choroidal vessels lack. In addition, other blood vessel characteristics, such as the blood vessel lumen size difference between retinal and choroidal vessels, and the vascular wall structure, could affect the signal peak time. Another finding is that the vessels injected with the higher concentration of gold nanoparticles achieved peak signal enhancement longer than the one injected with the lower concentration of gold nanoparticles due to higher accumulation of gold nanoparticles at the blood vessels wall over time after injection. This finding is consistent with a phenomenon as described by Fullstone *et al*.^63^. The authors described that the PA amplitude could be enhanced by the influence of particles at the vessel wall. Thus, the AuNP signal enhancement peaks between retinal after

AuNPs also exhibited strong OCT contrast *in vivo* due to enhanced scattering properties and improved visualization of small blood vessels. After IV injection, OCT signal contrast was increased by 45%. The generated high OCT intensities could be caused by combined backscattering of 900 nm light by hemoglobin (i.e., scattering coefficient = 7 cm^−1^ for hemoglobin)^64^ and scattering by the AuNPs in the vessels. The scattering spectrum of AuNPs is shown in Figure S5. For the current AuNPs used, the peak of the optical scattering is around 532-nm which is consistent with a previous study^65^. According to Fig. S2, the measured scattering coefficient of AuNPs at 900 nm is approximately 22 cm^−1^, which is about 5% scattering at 535 nm. To further enhance the optical scattering for 900-nm wavelength light used by OCT, the size and the shape of the AuNPs can be adjusted.

Another challenge for ocular imaging is to rapidly achieve high resolution images without damaging sensitive neural tissue. Therefore, the ocular imaging system must meet several requirements. 1) The illumination intensity must be below ANSI safety limit. Delori *et al*. and Organisciak *et al*. have demonstrated that the retina can be injured by higher intensity exposure, leading to thermal damage, thermoacoustic damage, and photochemical damage^66,67^. 2) High imaging speed is needed to avoid possible motion artifacts. Robinson *et al*. has reported that the eye has a very short fixation time of approximately 500 ms^68^. This motion can cause image blurring or image disruption. The described system has an integrated PAM and OCT imaging system for monitoring blood vessels in rabbits. The system has excellent lateral and axial resolution (i.e., 4.1 µm and 37.0 µm for PAM and 3.8 µm and 4.0 for OCT). When working on the PAM mode, this system could distinguish retinal and choroidal blood vessels using laser light energy of ~80 nJ, a safe dose that is only half of the ANSI safety limit (i.e., < = 160 nJ at 532 nm)^27,69^. The current system with a tunable OPO with a 1 kHz pulse repetition rate takes 65 seconds for a 3 × 3 mm raster-scan region on the retina with 256 × 256 pixels. This imaging speed is about 25 times faster in comparison with those based on mechanical scanning methods, for example the one reported by *Song et al*. showing an scanning time of about 25 min^70^. However, by switching to commercially available 10 MHz nanosecond pulse duration single wavelength lasers, this scan speed can likely be reduced to 6.5 ms. In addition, the use of AuNP conjugated to targeting agents can allow for molecular imaging to be achieved. To minimize the potential toxicity of metallic contrast agents for optical imaging, organic dyes could be investigated as a potential contrast agent for multimodal imaging with OCT. For example, Yoo *et al*. have demonstrated that the use of Cy7 fluorophore allowed enhancing OCT image contrast, leading to improving the visualization of artery and vessel walls^71^.

In summary, this is the first study demonstrating PEG-AuNPs can serve as a multimodal contrast agent for PAM and OCT *in vivo* in rabbits to visualize retinal and choroidal microvasculature. This is an important step in clinical translation since the rabbit eye is similar in size to that of humans. Imaging of microvasculature remains an important clinical target as neovascularization can lead to vision loss in numerous diseases, including macular degeneration and diabetic retinopathy. The fabricated PEG-AuNPs not only had good stability in physiological environments, but also were non-toxic to vascular endothelial cells at the experimented concentrations. PEG-AuNPs significantly augmented both OCT and PAM signal by 81% for PAM and 45% for OCT for the improved visualization of the retinal and choroidal microvasculature. The PEG-AuNPs–assisted PAM modality could distinguish single vessels at low laser irradiance of 80 nJ, which is below the ANSI safety limit (160 nJ) at 532 nm.

## Materials and Methods

### Chemical materials

All chemicals were used as received without further purification. Methoxy-PEG-thiol with a molar mass of 5000 g mol^−1^ (mPEG-SH5k) was purchased from Creative PEGWorks (Chapel Hill, NC, catalog # PLS-604). mPEG-SH5k was in powder form and dissolved in deionized (DI) water prepared using Milli-Q Academic water purification system (Billerica, MA) having an electric conductivity less than 0.7 μS cm^−1^. All solutions were freshly made as needed and used within twelve hours. The bulk gold target (16 mm long, 8 mm wide, 0.5 mm thick, and 99.99% purity) used in the laser ablation experiment was purchased from Alfa Aesar (Ward Hill, MA). EndoGRO were ordered from Vec Technologies (Rensselaer, NY, USA). Complete MCDB-131, trypsin–ethylenediaminetetraacetic acid (trypsin-EDTA), antibiotics/antimycotics, fetal bovine serum (FBS), phosphate-buffered saline (PBS) were purchased from Gibco BRL, Life Technologies (Grand Island, NY, USA). Heparin, epidermal growth factor (EGF), fibronection, tylosin, sodium bicarbonate, 3-(4,5-Dimethyl-2-thiazolyl)-2,5-diphenyl-2H-tetrazolium bromide (MTT), dimethyl sulfoxide (DMSO), Hoechst 33342, and propidium iodide (PI) were obtained from Sigma-Aldrich (Sigma, St. Louis, Mo, USA).

### Synthesis of PEG-AuNPs

PEGylated gold nanoparticles (PEG-AuNPs) were synthesized as multimodal contrast agents for PAM and OCT imaging. A detailed synthesis of PEG-AuNPs was reported by Bing Liu *et al*.^47^. In brief, PEGylated gold nanoparticles (PEG@AuNPs) were prepared from the raw colloidal AuNPs (Appendix A) with bare surfaces by simply mixing with a solution of mPEG-SH5k. For 50 mL stable colloidal solution of 20 nm gold nanoparticles with optical density (OD) of 1 at 520 nm (6.11 × 1011 nanoparticles/mL), 0.5 mL mPEG-SH5k solution with concentration of 1 milliMolar (mM) was added and mixed well. Treatment of raw AuNPs with this concentration of mPEG-SH5k ligand in water leads to rapid coating with the PEG molecules. After 10 minutes, an increase of the hydrodynamic size could be observed by characterizing with dynamic light scattering analysis. The mixture of AuNPs and mPEG-SH5k was kept undisturbed for 2 hrs at room temperature to enable sufficient conjugation of mPEG-SH5k molecules to the AuNPs via Au-S bonding. After the reaction, the nanoparticle dispersion was transferred into a 50 mL centrifugal tube and centrifuged at 8000 g for 1 hr, until a pellet was formed. The supernatant was removed and 50 mL DI water was added. This process was repeated two times to remove any unreacted PEG molecules. The final PEGylated AuNPs were collected and resuspended to an OD of 100 by adding DI water.

### Characterization of PEG-AuNPs

The synthesized colloidal AuNPs and PEG@AuNPs were characterized by an array of analytic instruments and techniques, including transmission electron microscopy (TEM), ultraviolet-visible (UV-Vis) spectroscopy, and dynamic light scattering (DLS). TEM was used to visualize the fabricated nanoparticles. Micrograph of the colloidal AuNPs were recorded using a TEM (JEOL 2010F, Japan) at an accelerating voltage of 100 kV and the median size of the nanoparticles was then calculated with Image J software (National Institute of the Health, Bethesda, MD, USA). To select the excitation wavelength for PAM, the absorption spectra of PEG-AuNPs was measured from 350 to 800 nm by using a spectrophotometer (UV-3600, Shimadzu Corp., Japan). DLS measurement with Zetasizer Nano ZS90 (Malvern Instruments, Malvern, Worcestershire, UK) was employed to measure hydrodynamic diameter of AuNPs before and after surface modification with mPEG-SH5k molecules. The infrared spectra of mPEG-SH5k, AuNPs, and PEG-AuNPs were measured using a PerkinElmer spectrum 100 Fourier transformed infrared spectroscopy (FTIR) spectrometer (PerkinElmer Inc., Waltham, MA) equipped with an attenuated total reflection (ATR) diamond. All measurements and processes were carried out at room temperature, approximately 21 °C.

## Cytotoxicity assessments

### Cell culture

Bovine retinal endothelial cells (BREC) were provided by the generous assistance of Dr. David Antonetti. The BREC cells were cultured as monolayers in 100-mm culture dishes. Prior to culturing, the culture plates were coated with fibronectin at a concentration of 1 µg/mL and kept for 1–4 hours at room temperature. Complete MCDB-131, which were supplemented with 10% FBS, 1.18 g sodium bicarbonate, 20 ng/mL EGF, 200 mg EndoGRO, 90 mg heparin, 1 mL tylosin and 10 mL antibiotics/antimycotics, were prepared as the culture medium for the BREC cells. The cells were incubated at 37 °C in humidified atmosphere of 5% CO_2_ and 95% air. The culture medium in the cell plates was changed every day. When the BREC cells in the culture dish reached 70–90% confluence, they were isolated by adding 3 mL of 0.25% trypsin-EDTA solution into dishes and incubated for 3 min. The harvested cells were then centrifuged at 500 rpm for 5 min.

### Biocompatibility of PEG-AuNPs

Biocompatible properties of PEG-AuNPs were evaluated on BREC cells. The cells were cultured in 96-well plates at an estimated density of 1 × 10^4^ cells/well and incubated for 24 hours at 37 °C in a humidified atmosphere of 5% CO_2_. Then, the culture medium was replaced with fresh medium containing PEG-AuNPs at various final concentrations (i.e., 0.5, 12.5, 25, 50, 100, 200, and 400 µg/mL). Control cells were used without using PEG-AuNPs. The cells were further incubated for 24 h and 48 h. The toxicity effect of PEG-AuNPs were evaluated by using a standard methyl tetrazolium (MTT) assay.

### Apoptosis assay (Hoechst and PI double staining)

To evaluate cell characteristics such as nuclear morphology, apoptosis, and necrosis, fluorescence microscopic analysis was performed by using Hoechst 33342 and propidium iodide (PI) double staining. The cells with homogeneously stained nuclei were considered viable, and the cells with chromatin condensation or fragmentation were considered apoptotic^72,73^. In contrast, necrotic cells were only stained with PI. Hoechst/PI double staining was employed on BREC cells. The cells were cultured in 33 mm µ-dish plates at 2 × 10^5^ cells/well and incubated for 24 h at 37 °C in a humidified atmosphere of 5% CO_2_. Then, the medium in the plates was discarded, and the cells were then treated with fresh media containing PEG-AuNPs at various final concentrations (i.e., 50, 100, and 200 µg/mL) and further incubated for 4 h. A control was prepared without incubation with nanoparticles. After the incubation time, the cultured cell plates were washed three times with PBS to remove unattached nanoparticles before double staining with Hoechst 33342 and PI fluorescent dyes. After washing, the cells were fixed with 4% formaldehyde and incubated for 20 min at 37 °C. Next, the cells were washed with cold PBS two times, and then, 300 µL of 10 µg/mL Hoechst solution was added to the cells and incubated for another 20 min at 37 °C in the dark environment. The cells were then washed two times with PBS, and 300 µL PI (10 µg/mL) was added. The sample was incubated for an additional 10 min at 37 °C. Finally, the stained cells were washed three times with cold PBS and captured with a Leica fluorescence microscope equipped with a DFC450C color digital camera (Leica DM 6000, Wetzlar, Germany).

### Statistical analysis

Statistical analysis was conducted using SPSS (Ver 22, IBM Corporation, Armonk, New York) to determine any significant difference between the two experimental groups. The Student’s t-test was applied to compare image contrast before and after injection. For non-parametric statistical analysis, Mann-Whitney U test was used to determine any significant difference between the two experimental groups. Data were expressed as mean ± standard deviation (SD). *P-*values of < 0.05 indicated statistical significance.

## Sample Preparations

### Phantom preparation

A group of phantom samples was prepared and used to examine PAM response of fabricated PEG-AuNPs. The phantom was made of silicone tube with an inner diameter of 0.30 mm and outer diameter of 0.64 mm (N = 6). The tubes were filled with different concentration of PEG-AuNPs suspension solution (i.e., 0 (saline), 0.5, 1, 2, 4, and 5 mg/mL) by a 30 gauge 1 mL insulin syringe (Covidien, MA, USA). To compare the photoacoustic signal between blood and AuNPs, AuNPs suspension was mixed with blood at a ratio of 1:1. The blood and mixed solution were injected into silicone tubes. Both the distal ends of each tube were sealed with optical adhesive. Prior to PAM, the phantoms were placed on the top of the coverslip. The coverslip was then placed in a degassed water bath to prevent any cavitation.

### Animal model preparation

All rabbit studies were employed under the guidelines of the ARVO (The Association for Research in Vision and Ophthalmology) Statement on the care and use of laboratory animals in Ophthalmic and Vision Research. The experimental protocol was approved by the Institutional Animal Care and Use Committee (IACUC) of the University of Michigan (Protocol numbers: PRO00006486 and PRO00008566, PI: Y. Paulus). New Zealand White rabbits that were 2–3 months old and weighed 1.8–2.8 kg were obtained from the Center for Advanced Models and Translational Sciences and Therapeutics (CAMTrasST) at the University of Michigan Medical School and used for the experiments. The Dutch Belted pigmented rabbits were purchased from Covance.

### Dual photoacoustic microscopy (PAM) and optical coherence tomography (OCT) imaging system

A custom-built dual modality PAM and OCT system (Figure S1) was used for the imaging studies^27,28^. PAM utilizes an optical parametric oscillator (OPO) pumped by a diode-pumped Q-switched Nd:YAG laser (NT-242, Ekspla, Lithuania, pulse repetition rate 1 kHz, duration 3–6 ns, tunable wavelength range 405–2600 nm). The laser light from OPO was perpendicularly reflected at the prism and then spread through a through a half-wave plate attenuator mounted on a motorized rotation stage; it was then focused, filtered, and collimated by a beam collimator. The beam collimator is composed of a focusing lens (focal length 250 mm), a pinhole (diameter 50 μm), and a collimating lens (focal length 30 mm). After collimation, the circular-shaped pattern was formed with a diameter of approximately 2 mm. The circular-shaped light was then split with a ratio of 90/10 (reflection/transmission). The transmitted portion was recorded by a photodiode for pulse-to-pulse laser energy monitoring. The reflected portion was successively deflected by a mirror and a dichroic mirror (DM) and raster-scanned by a two-dimensional galvanometer, which is a shared component with the spectral domain (SD)-OCT system. The scanned beam traveled through a telescope consisting of a scan lens (focal length 36 mm) and an ophthalmic lens (OL, focal length 10 mm). After travelling through the lenses, a laser beam with a diameter of approximately 2 mm was formed and was finally focused on the fundus by the rabbit eye optics. The laser light fluence on the eye used to acquire images was 80 nJ at 532 nm, which is half of the American National Standards Institute limit^27^. The ANSI maximum permissible exposure (MPE) for retinal exposure to single nanosecond pulses in the 400 to 700 nm spectral range is:

1 $${{\rm{MPE}}}_{{\rm{sp}}}={\rm{5.0}}\times {{\rm{10}}}^{-{\rm{7}}}{\rm{J}}{{\rm{.cm}}}^{-{\rm{2}}}$$

For the repetitive pulse limit:

2 $${{\rm{MPE}}}_{rp}={{\rm{n}}}_{total}^{-0.25}\,{{\rm{MPE}}}_{sp}$$

Within a laser spot of 20 um, there are at most two overlapping laser pulses (n_total_ = 2). Thus,

3 $${{\rm{MPE}}}_{{\rm{rp}}}={{\rm{n}}}_{{\rm{total}}}^{-{\rm{0.25}}}\,{{\rm{MPE}}}_{{\rm{sp}}}={{\rm{2}}}^{-{\rm{0.25}}}\,{{\rm{MPE}}}_{{\rm{sp}}}={\rm{4.2}}\times {{\rm{10}}}^{-{\rm{7}}}{\rm{J}}{{\rm{.cm}}}^{-{\rm{2}}}$$

The fluence on the cornea:

4 $${\rm{\Gamma }}=\frac{{\rm{{\rm E}}}}{{\rm{A}}}=\frac{{{\rm{E}}}_{{\rm{p}}}}{{\rm{\pi }}{(\frac{{\rm{D}}}{{\rm{2}}})}^{{\rm{2}}}}={\rm{0.026}}\,{{\rm{E}}}_{{\rm{p}}}$$

where Ep is the maximum permissible single laser pulse energy on the retina (J).

For a single-pulse MPE calculation, assuming a beam diameter matched to a fully dilated pupil (7 mm). The maximum permissible single laser pulse energy on the retina is

5 $${{\rm{\Gamma }} < \mathrm{MPE}}_{{\rm{rp}}}={\rm{4.2}}\times {{\rm{10}}}^{-{\rm{7}}}{\rm{J}}{{\rm{.cm}}}^{-{\rm{2}}}$$

Form (3), (4) and (5), the max energy for single pulse exposure is

6 $${\rm{E}}=\frac{{\rm{\Gamma }}\,}{{\rm{0.026}}}=\frac{{\rm{4.2}}\times {{\rm{10}}}^{-{\rm{7}}}}{{\rm{0.026}}}={\rm{160}}\,{\rm{nJ}}$$

To detect the laser-induced acoustic signals, a custom-made needle-shaped ultrasound transducer with a central frequency of 27.0 MHz (Optosonic Inc., Arcadia, CA, USA) with a center frequency of 27.0 MHz, two-way −6dB bandwidth 60% was mounted in contact with the conjunctiva of the central visual axis and aligned to enable accurate alignment with laser light. The axial and transverse resolutions were 37.0 and 4.1 µm, respectively. The received analog photoacoustic signals were filtered and amplified using a low-noise amplifier (AU-1647, L3 Narda-MITEQ, NY). After the amplification, the signals were converted into digital signals and recorded using a high-speed digitizer at a sampling rate of 200 MS/s (PX1500-4, Signatec inc., Newport Beach, CA). The recorded data was then used to reconstruct two-dimensional (2D) or three-dimensional (3D) images of the eye blood vessels. 2D depth-sensitive PAM images were acquired by implementing horizontal scanning lines along the x-axis. For 2D image reconstruction, each sample was scanned along x- and y-directions using an optical-scanning galvanometer with a resolution of 2.5 × 5 µm^2^, while scanning depth (z-direction) was fixed at the focal depth of the imaging transducer. For a 1.5 × 1 mm^2^ field of view, the acquisition time was approximately 60 s. In addition, by performing faster scanning along the y-axis, the volumetric PA images were obtained accordingly. To assess the potential of the fabricated nanoparticles for the enhanced PA image contrast, the image contrast, which was defined as the difference between the PA signal of the targeted area and its background, was estimated from the reconstructed images as described in previous studies^11,12,74^. Additionally, to visualize the margin of the blood vessel, 3D image reconstruction was performed as a post image processing analysis. The 3D image reconstruction was performed on a set of 256 A-scan images with a gap of 2.5 µm between the consecutive slides. These images were selected, aligned, and combined using Amira software. Further post-processing was performed on the 3D image to improve visualization of the newly formed blood vessels margin.

Optical Coherence Tomography (OCT) was performed from a commercially available OCT system (Ganymede-II-HR, Thorlabs, Newton, NJ) by adding the ocular lens after the scan lens and a dispersion compensation glass in the reference arm^27,28^. A combination of two super luminescent diodes with center wavelengths of 846 nm and 932 nm was used to excite the tissue. The lateral and axial resolutions are 4 µm and 3 µm, respectively. The OCT light source was coaxially aligned with the PAM system. Thus, OCT can be used as guided PAM and help interpret PAM results. Fundus photography was performed using the Topcon TRC 50EX fundus camera (Topcon Corporation, Tokyo, Japan).

### *In vivo* PAM/OCT for retinal and choroidal vessels evaluation

Retinal and choroidal blood vessels were evaluated by dual PAM/OCT pre and post intravenous (I.V.) injection of the synthesized nanoparticles. All PAM and OCT analyses of the retinal and choroidal blood vessels with the integration of nanoparticles for comparison of signal enhancement pre- and post-administration were conducted as blinded and randomized experiments. 13 New Zealand White rabbits (~2.5 kg) and 3 Dutch Belted pigmented rabbits (~2.0 kg) were used for all the imaging studies to detect the margin of blood vessels. Prior to the experiments, animal state, mucous membrane color, temperature, heart rate, and respiratory rate were monitored and recorded as a general procedure by using a pulse oximeter (Smiths Medical, MN, USA). Then, a mixed solution of ketamine (40 mg/kg IM, 100 mg/mL) and xylazine (5 mg/kg IM, 100 mg/mL) was used to fully anesthetize each rabbit model with an intramuscular application. To sustain anesthesia during the *in vivo* experiments, a vaporized isoflurane anesthetic (Surgivet, MN, USA) was applied to provide 1 L/min oxygen and 0.75% isoflurane. The pupils of rabbit were diluted using tropicamide 1% ophthalmic and phenylephrine hydrochloride 2.5% ophthalmic. Topical tetracaine 0.5% was instilled in the eye for topical anesthesia in addition to lubricant (Systane, Alcon Inc., TX, USA) to prevent dehydration of the cornea. The PAM and OCT images of the retinal and choroidal vessels were obtained before and after the I.V injection of AuNP (0.8 ml, 2 and 5 mg/ml). A water-circulating blanket (TP-700, Stryker Corporation, Kalamazoo, MI) was used to maintain the temperature of the rabbit during the experiment. After the anesthetic injection, the rabbits were positioned on the imaging platform, and the areas of interest were monitored by the fundus camera. The head and body of the rabbit were placed on different high performance, custom-made stabilization platforms to minimize breathing and other motion artifacts. The rabbit vessels were first imaged with the OCT system. Then, an ultrasound transducer was mounted in the eye chamber, allowing it to move freely in 3D while not applying any physical pressure on the rabbit eyes. The ultrasound gel was sandwiched between the eye and the transducer. Then, the targeted regions were selected by the fundus camera and imaged with PAM. After acquiring the control PAM image, PEG-AuNPs (0.8 mL, 2 and 5 mg/mL) were intravenously injected into the rabbit with a 1 mL syringe, 27-gauge needle in the marginal ear vein. PAM images of the injected rabbit were performed immediately after injecting nanoparticles. After the *in vivo* experiments, vitals including mucous membrane color, heart rate, respiratory rate, and rectal temperature were monitored and recorded until the rabbit fully recovered. Then, the rabbit was immediately returned to its rack and monitored the body weight every day for 7 days. In addition, to distinguish the enhancement photoacoustic signal between the normal vessels and the injected vessels, image matching and subtraction algorithms were performed as post-image processing using Matlab software (MathWorks, Massachusetts, USA) (Appendix B). The three dimensional structure of blood vessels were also reconstructed to visualize the structure and estimate the detected diameter of blood vessels by using Amira software.

### Histological analysis

To evaluate the toxicity of PEG-AuNPs for *in vivo* PAM and OCT, rabbits were euthanized seven days after intravenous infusion of AuNP, and the eyes and other vital organs were extracted for histological analysis. Rabbits were euthanized by injection of intravenous injection of pentobarbital (Beuthanasia-D, 0.22 mg/kg I.V., 50 mg/mL) (Intervet Inc., Madison, NJ, USA). The eye and different tissues from the treated group and control group were removed aseptically from the euthanized rabbits. The isolated samples were fixed in 10% neutral buffered formalin (VWR, Radnor, PA) for a minimum of 48 h. The fixed tissues were cross-sectionally cut in 5 mm sections and embedded in paraffin. Subsequently, the paraffin-embedded tissues were sliced to a thickness of 4 µm and stained with hematoxylin and eosin (H&E) and terminal deoxynucleotidyl transferase dUTP nick end labeling (TUNEL) assay using a Leica autostainer XL (Leica Biosystems, Nussloch, Germany) under standard conditions. The slides were observed by using a Leica DM600 light microscope (Leica Biosystems, Nussloch, Germany) to detect apoptotic or necrotic cells.

### Biodistribution of AuNPs

To investigate the biodistribution of PEG-AuNPs in the rabbit after 7 days of sample administration, the amount of gold in blood and organs (heart, lung, kidney, liver, and spleen) was determined. The blood and tissue samples were collected from the euthanized animal. The blood samples were stored in a refrigerator at 4 °C. The tissues were briefly washed with PBS and dried with blotting paper. Then, the isolated organs were weighed accurately and were stored in a freezer at temperature of −40 °C. The collected samples were processed as described in Takeuchi and Simpson *et al*. with modifications^53,75^. Briefly, 2 mL of the fluid sample or 0.2 g of organ sample was dissolved at elevated temperature (100 °C) in a 10 mL concentrated 67% nitric acid for 30 minutes. Subsequently, 30 mL hydrochloric acid was added and then heated to dryness for 3 more hours until the mixed solution reached 5 mL. Melted solutions were filtered with 0.22 µL cellulose nitrate syringe filter (Merc Millipore Ltd., Co. Cork, Ireland) and dissolved in 5 mL deionized water. All obtained samples were then analyzed on inductively coupled plasma mass spectrometer (ICP-MS) for gold content using a standard calibration. A blank and three standards were used for calibration. Analyses of diluted blood and tissue samples were performed on an SQ-ICP-MS ((iCAP™ RQ ICP-MS, Thermo Fisher Scientific, Bremen, Germany) at Michigan State University.

## Supplementary information

Visualization 3

Visualization 1

Visualization 2

Supplementary Info

Dataset
