# Supplementary material for: Contrast Agent Enhanced Multimodal Photoacoustic Microscopy and Optical Coherence Tomography for Imaging of Rabbit Choroidal and Retinal Vessels in vivo
Source: Sci Rep. 2019 Apr 11;9:5945. doi: 10.1038/s41598-019-42324-5 (PMC6459908; doi:10.1038/s41598-019-42324-5)
Supplement: Supplementary file 4 — Supplementary Info [file 41598_2019_42324_MOESM4_ESM.docx]

**Contrast Agent Enhanced Multimodal Photoacoustic Microscopy and Optical Coherence Tomography for Imaging of Rabbit Choroidal and Retinal Vessels *in vivo***

**Van Phuc Nguyen^1^, Yanxiu Li^1,6^, Wei Qian^4^, Bing Liu^4^, Chao Tian^1^, Wei Zhang^2^, Ziyi Huang^3^, Arjun Ponduri^1^, Madison Tarnowski,^1,2^ Xueding Wang^2,5^, and Yannis M. Paulus^1,2* ­­­^**

^1^Department of Ophthalmology and Visual Sciences, University of Michigan, Ann Arbor, MI 48105, USA

^2^Department of Biomedical Engineering, University of Michigan, Ann Arbor, MI 48105, USA

^3^Department of Electrical Engineering and Computer Science, University of Michigan, Ann Arbor, MI 48105, USA

^4^IMRA America Inc, Ann Arbor, MI 48105, USA

^5^Department of Radiology, University of Michigan, Ann Arbor, MI 48105, USA

^6^Department of Ophthalmology, Xiangya Hospital, Central South University, Changsha, Hunan 410008, China

^*^Corresponding Authors:

Yannis M. Paulus, M.D., F.A.C.S.

Department of Ophthalmology and Visual Sciences

Department of Biomedical Engineering

University of Michigan

1000 Wall Street

Ann Arbor, MI 48105, USA

Email Address: ypaulus@med.umich.edu

**Supplementary Information**


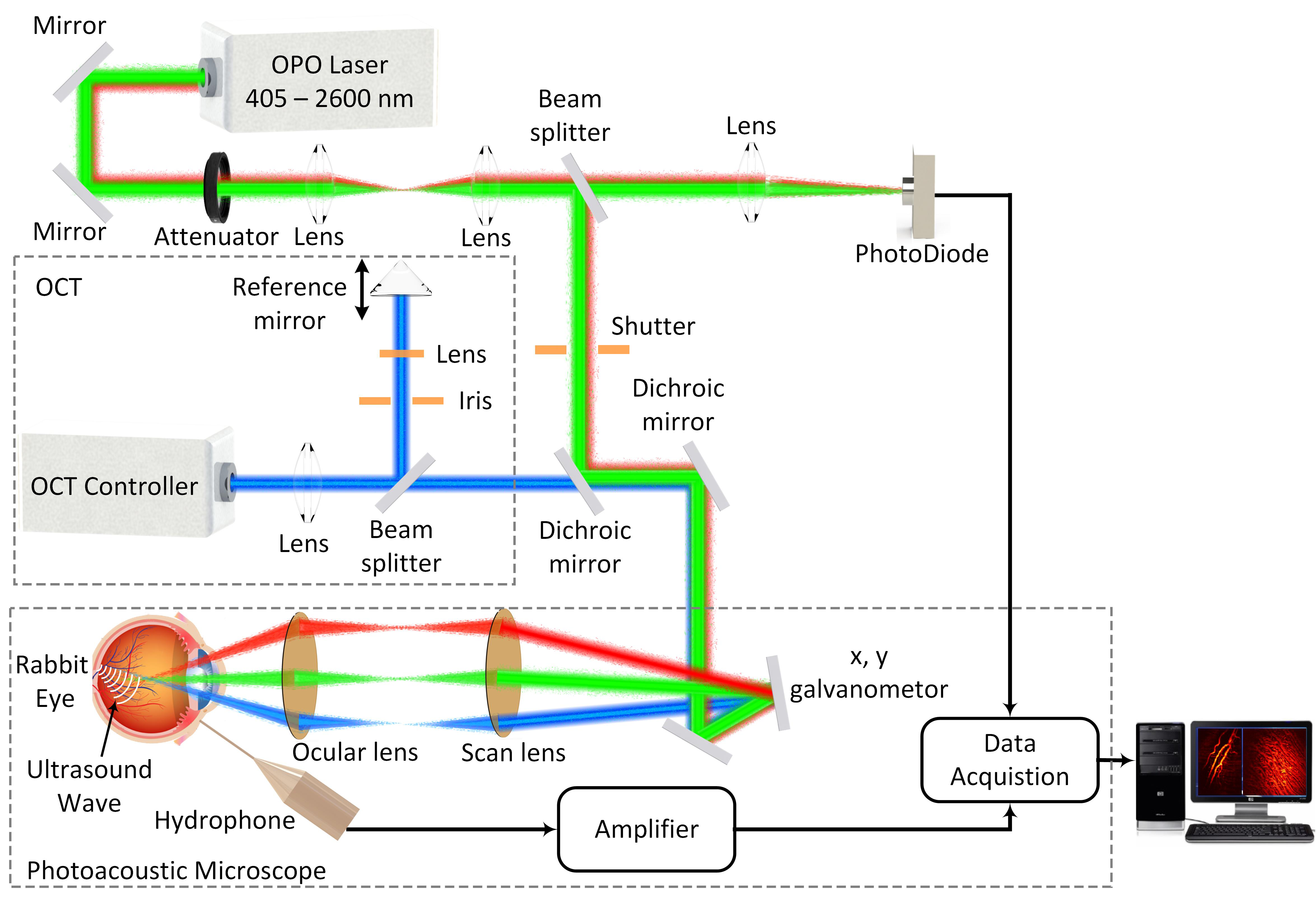


**Figure S1. S**chematic of the multimodal molecular ocular system with integrated OCT and photoacoustic microscopy (PAM). A pulse laser beam with a wavelength of 532 nm (PAM) or 905 nm (OCT) was delivered and focused onto the retina. The laser-induced ultrasound signal was recorded by a needle hydrophone and used to reconstruct PAM images. The reflected OCT light interferes with the reference light, and the interference intensity spectra was detected by a spectrometer. As the retina was scanned by a galvanometer, the 3D volumetric rendering was visualized.

**
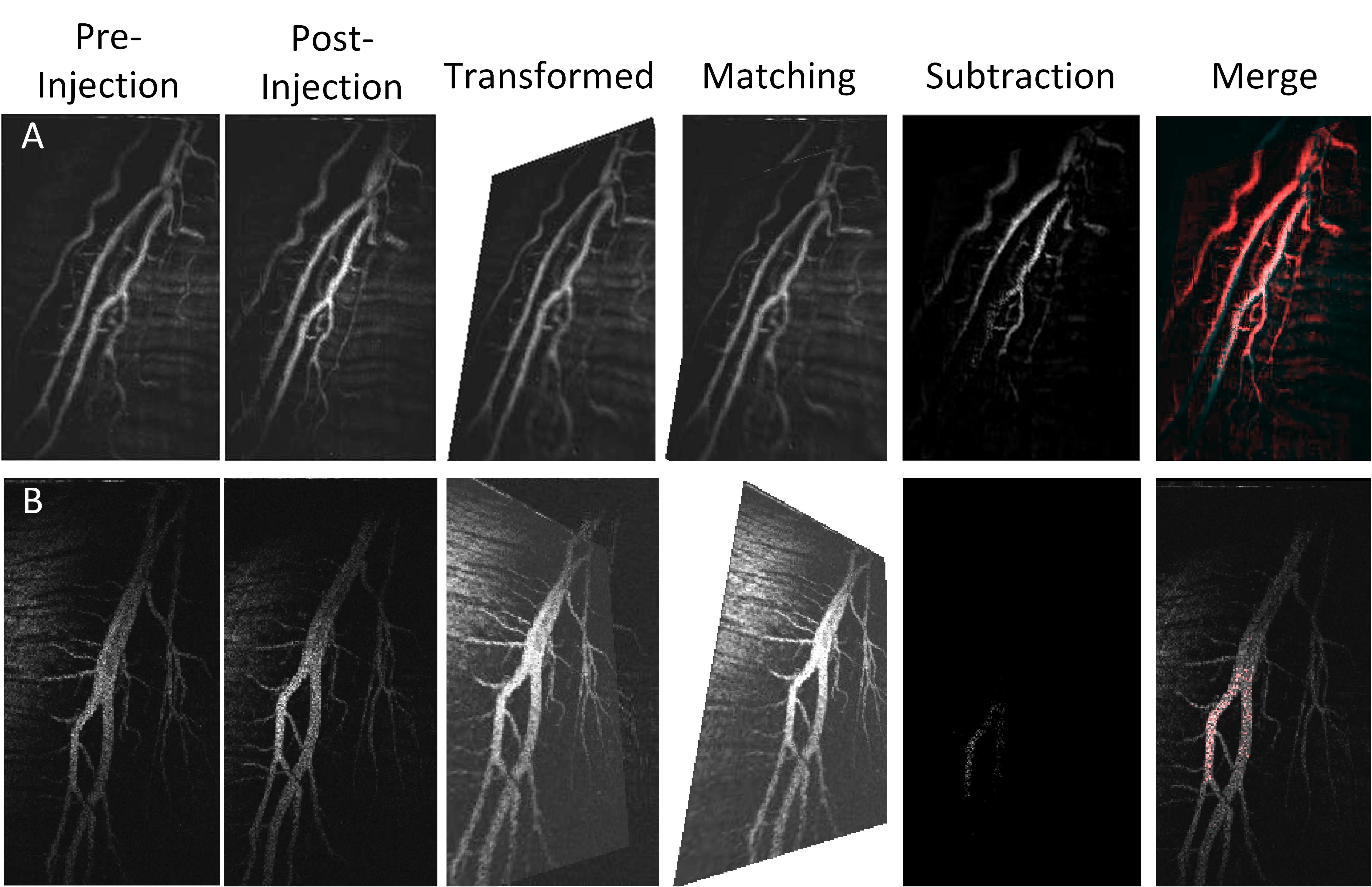
**

**Figure S2.** Image subtraction for PAM images of retinal blood vessels before and after injection of AuNPs. (A) Result for I.V. administration 0.8 mL AuNPs at 2 mg/mL concentration. (B) Result for I.V. administration 0.8 mL AuNPs at 5 mg/mL concentration. Due to motion artifact during in vivo experiment, image transformation was performed to co-register the pre- and post-injection images. The merging images show the enhanced signal after injection. Note that red color indicates the enhanced PA signals.


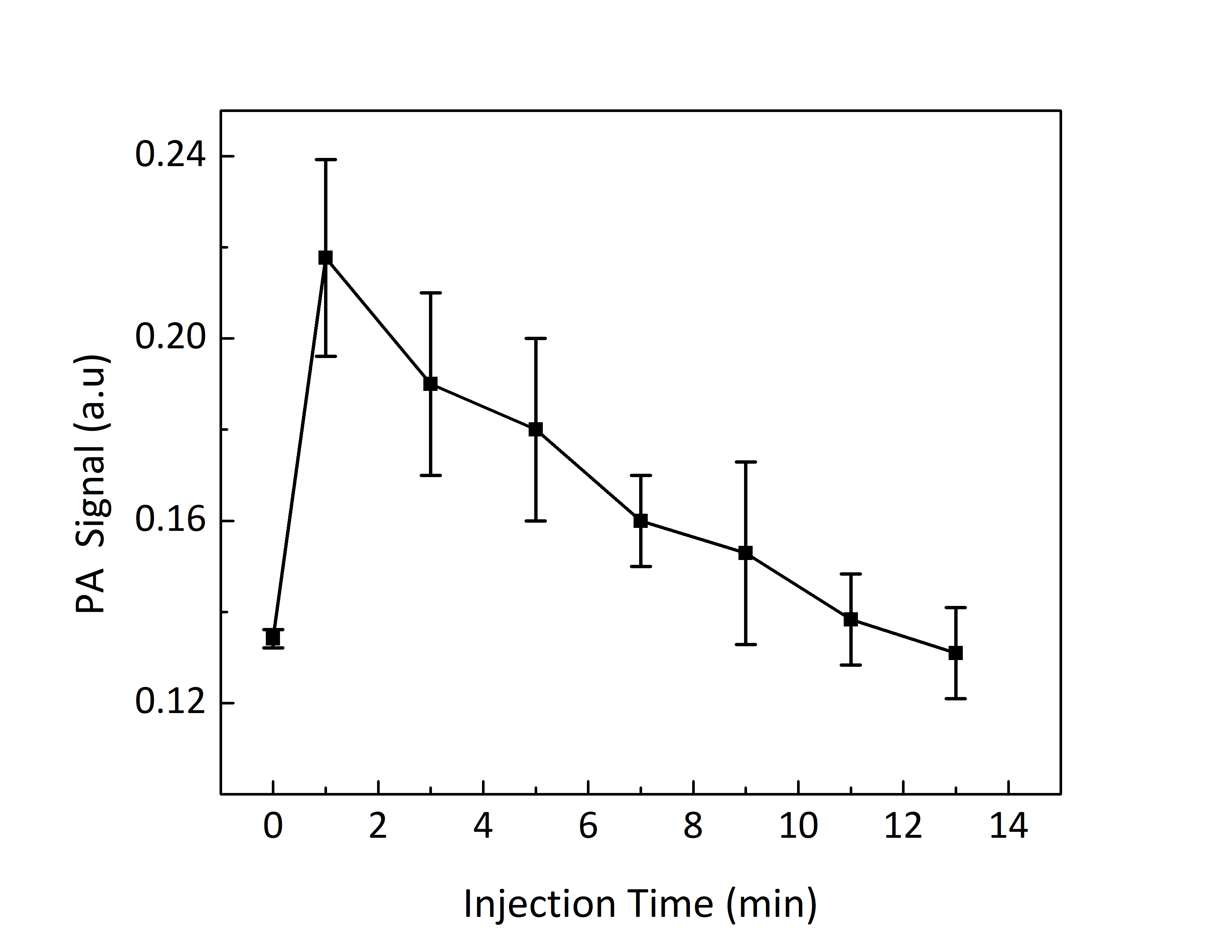


**Figure S3.** Quantitative analysis PA signals at different times on pigmented rabbit retinal vessel before and after injection of PEG-AuNPs (*p < 0.05 and N = 3).

**
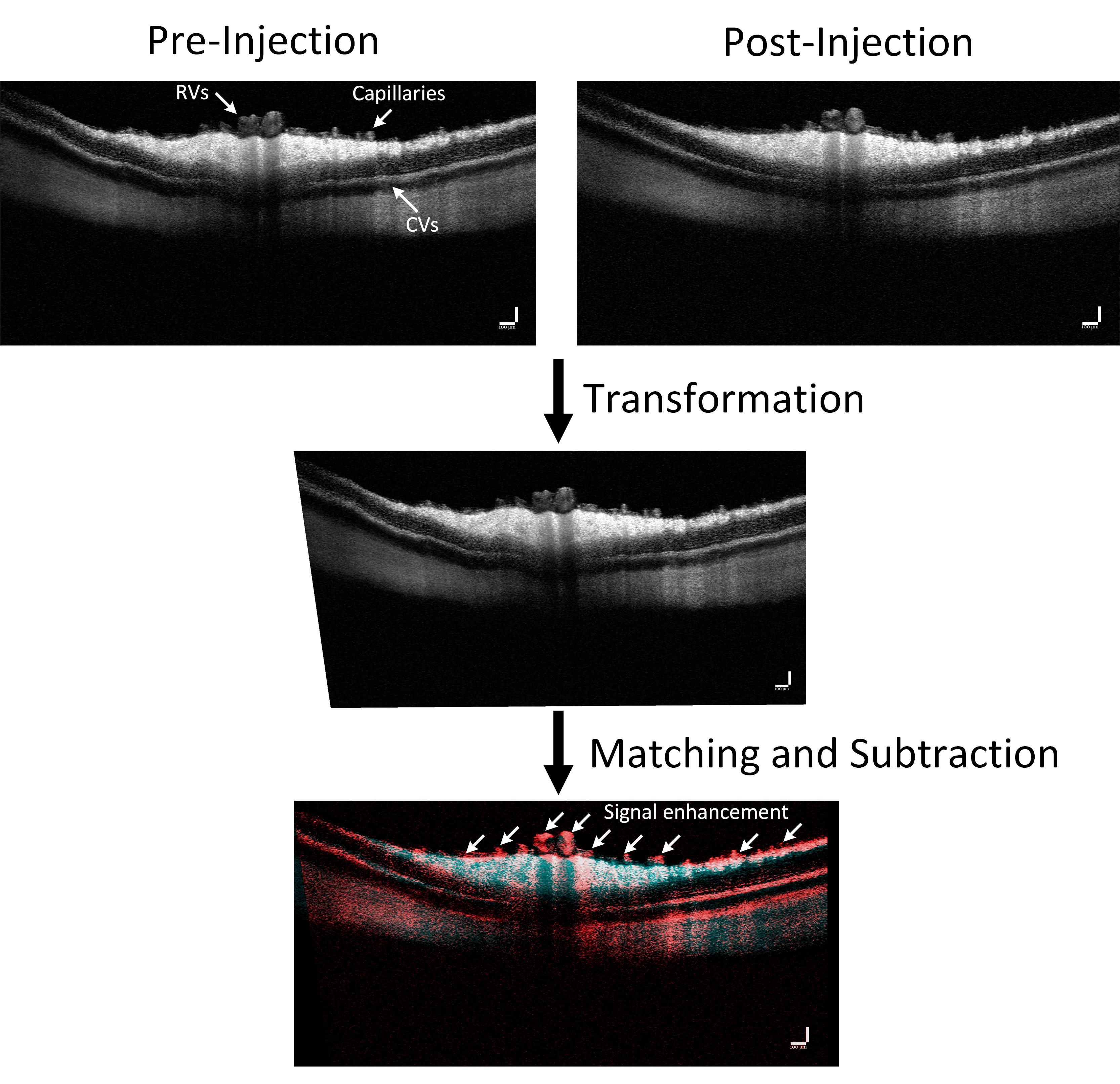
**

**Figure S4.** Post-OCT image transformation, matching, and subtraction of retinal vessels before and after intravenous administration of 0.8 ml AuNPs at the concentration of 5 mg/mL. Two original OCT image pre- and post-injection was transformed to co-register the image features, resulting in minimizing the differences of image features within each image. The pre-injection image was set as background and subtracted from the post-injection image. The subtracted image showed the enhanced OCT intensity signal after injection. White arrows indicate the signal enhancement.


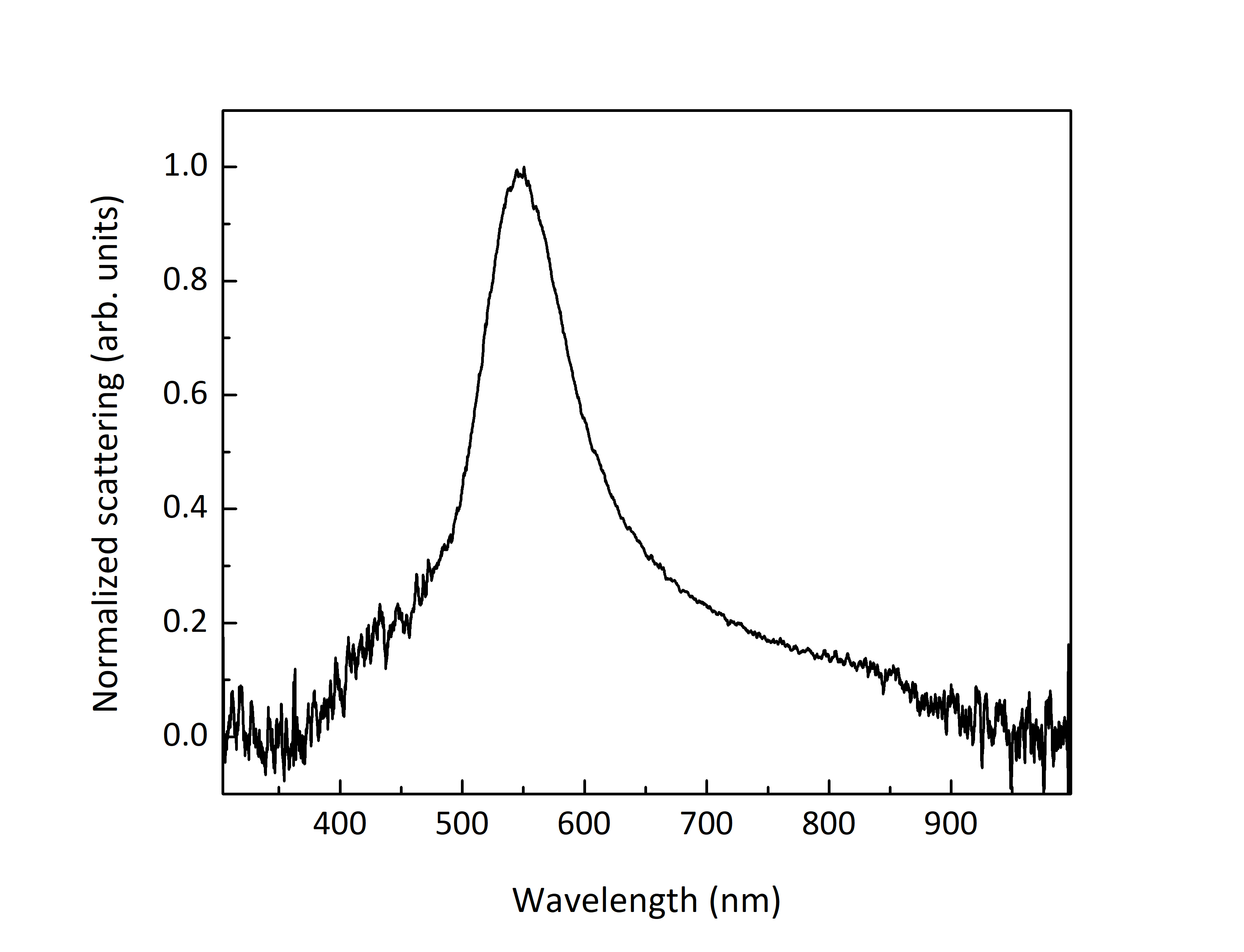


**Figure S5**. Calculated scattering spectroscopic of AuNPs by nonlinear method. It can be observed that peak scattering occurred at the wavelength of 550 nm, whereas smaller scattering occurred at longer wavelengths.

**Appendix**

**A. Synthesis of AuNPs by Femtosecond Laser Method**

Colloidal AuNPs with bare surfaces used in the present study were physically generated by femtosecond pulse laser ablation of a gold target in flowing deionized water (Figure S5). The ytterbium-doped femtosecond fiber laser (FCPA μJewel D-1000, IMRA America, Ann Arbor, MI) operating at 1.045 μm delivered pulsed laser at a repetition rate of 100 kHz with 10 μJ pulse energy and 700 fs pulse duration. The emitted laser beam was first focused by an objective lens, and then reflected by a scanning mirror to the surface of the bulk gold target, which was submerged in flowing deionized water (18 MΩcm). The size of the laser spot on the gold target was estimated to be 50 μm and its position was precisely controlled by the scanning mirror. During process, a translation stage was employed to produce relative movements between the laser beam and the gold sample. The generated nanocolloids were stably suspended in water and did not require any dispersants, surfactants or stabilizers to maintain their stability, which facilitates subsequent surface modification process. After aging the generated colloidal Au NPs for a couple of days, the top, clear, red solution was collected for use.


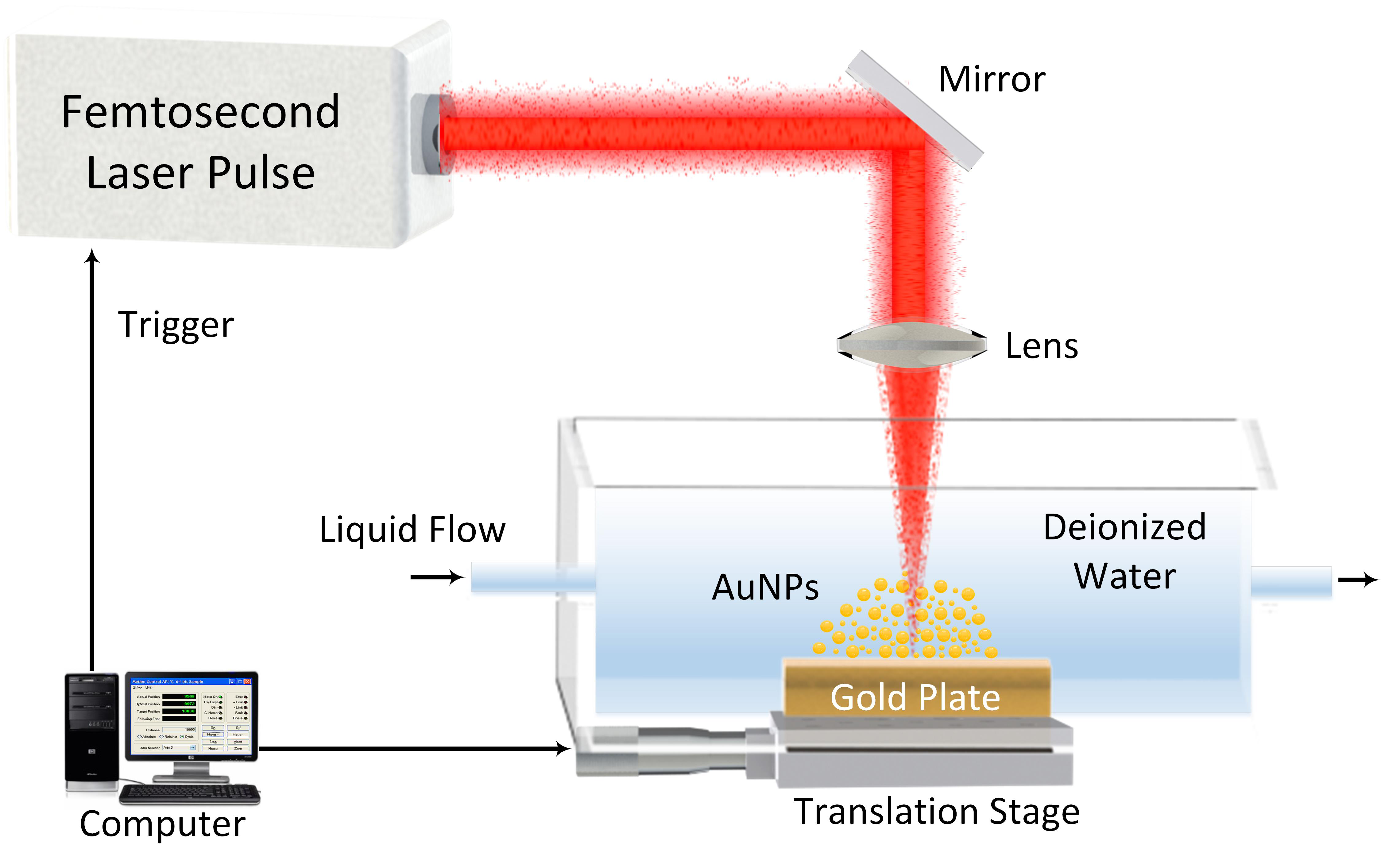


**Figure S6.** Colloidal AuNPs generation by femtosecond laser ablation of a gold target in flowing deionized water. This figure was adapted with permission from reference of Qian *et al*.[^1^](#_ENREF_1)

1. **Post Imaging Subtraction Algorithm**

Photoacoustic microscopy and optical coherence tomography are used to detect the position of absorbers and scatterers within the tissues[^2-5^](#_ENREF_2). To distinguish the enhanced PA and OCT intensity signals, image subtraction algorithm was carried out on the acquired images of retinal blood vessels after injection PEG-AuNPs. The image subtraction algorithm was performed by using Matlab (Mathworks, USA). The result was illustrated in Figure S1 and S2. In the last few decades, medical image processing has been investigated to find difference or changes between two mostly identical images by a number of researchers and clinicians. For example, digital-subtraction angiography (DSA) is the computer-assisted radiographic visualization of the carotids and cerebral vessels with a minimal view of background tissues[^6^](#_ENREF_6). In brief, the first PAM image acquired before injection of gold nanoparticles as contrast agents is used to generate a digital mask that is subtracted from the PAM image acquired after nanoparticle administration. The subtracted images were implemented as following:

First, image transformation was performed to find the matching point between the shape of images pre- and post I.V injection. The image transformation was employed by using transformation matrix[^7^](#_ENREF_7):

$\begin{matrix} \lambda\left[\begin{matrix} x' \\ y' \\ 1 \end{matrix}\right]=HX \\ =\left[\begin{matrix} h_{11} & h_{12} & h_{13} \\ h_{21} & h_{22} & h_{23} \\ h_{31} & h_{32} & h_{33} \end{matrix}\right]\left[\begin{matrix} x \\ y \\ 1 \end{matrix}\right] \end{matrix}$ (1)

where λ is arbitrary coefficient.

After transformed, the image before injection was subtracted, resulting in creating a highlight of the signal enhancement.

**References:**

1 Qian, W., Murakami, M., Ichikawa, Y. & Che, Y. Highly efficient and controllable PEGylation of gold nanoparticles prepared by femtosecond laser ablation in water. *The Journal of Physical Chemistry C* **115**, 23293-23298 (2011).

2 Vakoc, B. J. *et al.* Three-dimensional microscopy of the tumor microenvironment in vivo using optical frequency domain imaging. *Nature medicine* **15**, 1219-1223 (2009).

3 Wang, L. V. Multiscale photoacoustic microscopy and computed tomography. *Nature photonics* **3**, 503-509, doi:10.1038/nphoton.2009.157 (2009).

4 Zerda, A. *et al.* Optical coherence contrast imaging using gold nanorods in living mice eyes. *Clinical & experimental ophthalmology* **43**, 358-366 (2015).

5 Nguyen, V. P. *et al.* Doxorubicin-fucoidan-gold nanoparticles composite for dualchemo-photothermal treatment on eye tumors. *Oncotarget* **8**, 113719-113733 (2017).

6 Covey, A. M., Brody, L. A., Maluccio, M. A., Getrajdman, G. I. & Brown, K. T. Variant hepatic arterial anatomy revisited: digital subtraction angiography performed in 600 patients. *Radiology* **224**, 542-547 (2002).

7 Gonzalez Rafael, C., Woods Richard, E. & Eddins Steven, L. Digital image processing using MATLAB. *Editorial Pearson-Prentice Hall. USA* (2004).

**Media:**

**Visualization 1:** 3D image reconstruction of the retinal blood vessels pre- and post-injections of 0.8 ml gold nanoparticles at concentration of 2 mg/ml.

**Visualization 2:** 3D volumetric rendering of the retinal blood vessels pre- and post-injections of 0.8 ml gold nanoparticles at concentration of 5 mg/ml.

**Visualization 3:** 3D volumetric rendering of the choroidal blood vessels pre- and post-injections of 0.8 ml gold nanoparticles at concentration of 2 mg/ml.
